# Reprogramming Tumorigenesis and the Tumor Microenvironment with Flavokawains

**DOI:** 10.3390/cancers18142211

**Published:** 2026-07-09

**Authors:** Nath Pampita, Babu Santha Aswani, Bandari BharathwajChetty, Sameena Lone, Mangala Hegde, Sunil C. Kaul, Kazumi Hirano, Renu Wadhwa, Ajaikumar B. Kunnumakkara

**Affiliations:** 1 Cancer Biology Laboratory, Department of Biosciences and Bioengineering, Indian Institute of Technology Guwahati (IITG), Guwahati 781039, Assam, India; 2 AIST-INDIA DAILAB, National Institute of Advanced Industrial Science and Technology (AIST), Tsukuba 305-8565, Japan

**Keywords:** tumorigenesis, tumor microenvironment, phytochemicals, flavokawains, natural compounds

## Abstract

Cancer remains one of the most frightening global health challenges, contributing substantially to morbidity and mortality across diverse populations. In recent years, naturally derived compounds have attracted considerable attention due to their potential therapeutic efficacy and fewer adverse effects. Among these, the flavokawain subclass of chalcones, comprising Flavokawains A, B, and C, obtained from various plant sources, has emerged as a promising group of bioactive phytochemicals exhibiting a broad spectrum of pharmacological activities, with notable anticancer potential. This review critically compiles and evaluates the existing preclinical evidence regarding the anticancer mechanisms of flavokawains across various cancer models. It was found that these compounds have significant potential to inhibit cancer cell proliferation, induce apoptosis, disrupt cell-cycle progression, and modulate multiple molecular pathways implicated in tumorigenesis, including phosphoinositide 3 kinase/Akt/mammalian target of rapamycin (PI3K/Akt/mTOR), nuclear factor kappa-light-chain-enhancer of activated B cells (NF-κB), extracellular-signal regulated kinase/c-Jun N-terminal kinase/mitogen-activated protein kinase (ERK/JNK/MAPK) and so on. Importantly, flavokawains exert significant modulatory effects within the tumor microenvironment by suppressing angiogenesis through downregulation of vascular endothelial growth factor (VEGF) and Angiopoietin-1 (Ang-1), attenuating epithelial-mesenchymal transition via restoration of E-cadherin and suppression of vimentin and Snail1, inhibiting matrix metalloproteinase (MMP)-mediated extracellular matrix remodeling, and disrupting cancer stem cell (CSC)-supportive niches. Preclinical toxicity profiles suggest a favorable safety margin, though further investigation is required to fully elucidate their therapeutic index. Due to their multifaceted mechanisms of action and selective cytotoxicity toward cancer cells, flavokawains are considered promising preclinical candidates for development as adjuncts or alternatives to conventional chemotherapeutic agents.

## Simple Summary

Cancer continues to be one of the leading causes of death worldwide, and despite advances in treatment, many patients face limited options, significant side effects, and high costs. There is an urgent need for safer and more effective therapies. Flavokawains, a class of naturally derived plant compounds, have attracted growing attention for their potential medicinal properties. Growing laboratory evidence suggests that these compounds can kill cancer cells, inhibit their growth, and prevent their spread, while exhibiting relatively low toxicity toward normal cells. This review critically evaluates available preclinical research on the anticancer properties of flavokawains across various cancer types. By summarizing how these compounds work at the molecular level and assessing their safety profiles, this review aims to highlight their potential as future cancer treatments or as supplements to existing therapies, thereby guiding researchers and clinicians toward promising new directions in cancer drug development.

## 1. Introduction

Cancer remains a major global health burden, exerting profound physical, emotional, economic, and social impacts on patients, and currently stands as the second leading contributor to mortality worldwide [1,2,3,4]. The GLOBOCAN 2022 reported an estimated global burden of nearly 20 million new cancer diagnoses alongside 9.7 million fatalities [5]. In addition, global cancer cases are projected to exceed 35.3 million by 2050, reflecting a 76.6% increase in incidence rates [6]. While conventional interventions, including surgery, radiotherapy, and chemotherapy, are widely utilized, and newer modalities like immunotherapy and gene therapy have emerged, these treatment modalities are often constrained by substantial limitations and considerable financial burden, collectively diminishing patients’ quality of life [7,8,9,10,11,12]. Therefore, it is imperative to develop innovative, highly efficacious and safer anticancer therapeutics that can address the current limitations of conventional treatments [13].

Natural compounds have garnered substantial recognition in modern pharmacology owing to their remarkable chemical diversity, relatively low toxicity, multi-target activity, and widespread availability, making them an indispensable reservoir for drug discovery and the development of efficacious therapeutics [14,15,16]. Approximately 41% of the anticancer drugs have been derived from natural compounds [17]. Furthermore, the use of phytochemicals for cancer prevention is a promising and effective strategy to reduce cancer risk [18,19]. This review critically evaluates the anticancer potential of flavokawains, their biological properties, toxicity profiles, impacts on key tumor microenvironment (TME)-associated processes and explores future perspectives based on a range of preclinical studies.

## 2. Selection Criteria

A literature search was performed in PubMed, using “flavokawain” as the primary keyword. This initial search identified 140 records. Titles and abstracts were screened for relevance, followed by full-text evaluation against predefined inclusion and exclusion criteria. Publications such as book chapters, errata, editorials, narrative reviews, and non-English articles were excluded. After this selection process, a total of 53 peer-reviewed studies published up to 31 January 2026 met the inclusion criteria and were included in this review.

## 3. Chemical Nature of Flavokawains

Flavokawains exist in three distinct forms, namely Flavokawain A (FKA), Flavokawain B (FKB), and Flavokawain C (FKC). FKA, chemically identified as 2′-hydroxy-4,4′,6′-trimethoxychalcone, is a yellow crystalline chalcone derivative (molecular formula: C_18_H_18_O_5,_ molecular weight: 314.3 g/mol, and melting point: ~113 °C) [PubChem CID: 5355469] [20]. FKB, known as 4′,6′-dimethoxy-2′-hydroxychalcone, is a related trans-chalcone, a yellow crystalline derivative bearing two methoxy groups at the 4′ and 6′ positions and a hydroxy group at the 2′ position. It has a molecular formula of C_17_H_16_O_4_, a molecular weight of 284.31 g/mol, and a lower melting point of 91 °C [PubChem CID: 5356121]. FKC or 2′,4-dihydroxy-4′,6′-dimethoxychalcone is also a yellow crystalline compound like other flavokawains (molecular formula: C_17_H_16_O_5,_ molecular weight: 300.30 g/mol, and melting point of 194–195 °C) [PubChem CID: 6293081] [20]. Flavokawains exhibit poor aqueous solubility but dissolve readily in organic solvents such as dimethyl sulfoxide (DMSO) [21,22,23,24] (Table 1).

The three flavokawains share identical A-ring substitution patterns; however, their structural diversity lies in the B-ring substitution, which fundamentally influences their physicochemical characteristics and biological potencies. FKB, possessing an unsubstituted B-ring, displays the highest electrophilicity, conferring superior cytotoxic capabilities. FKA, with a 4-methoxy substitution on the B-ring, exhibits high selectivity toward cancer cells coupled with moderate potency and favorable safety attributes. FKC, bearing a 4-hydroxy B-ring group, demonstrates stronger hydrogen-bonding capacity, resulting in improved bioavailability while maintaining high selectivity and reduced hepatotoxic potential (Table 1). Consequently, these structural variations among the three flavokawains result in differential ADME (absorption, distribution, metabolism, excretion) profiles and binding affinities, which collectively determine their distinct anticancer mechanisms and therapeutic applicability. While all three compounds induce apoptosis and trigger G2/M cell-cycle arrest across various cancer models, their varying potencies and selectivity profiles suggest complementary roles in cancer therapeutics, with each compound offering unique advantages for different clinical applications and treatment strategies.

**Figure 1:**
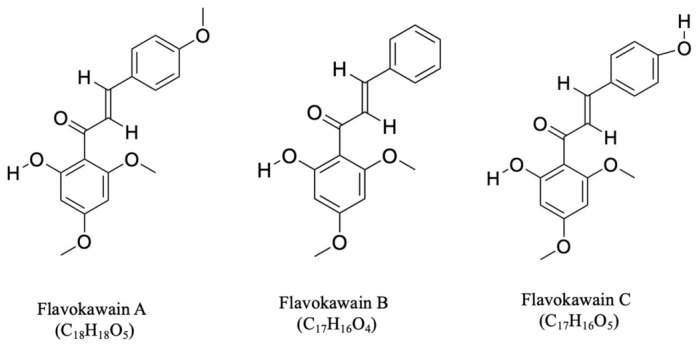
The chemical structures of the principal flavokawains-Flavokawain A (FKA), Flavokawain B (FKB), and Flavokawain C (FKC). These compounds belong to the chalcone class of flavonoids. Structural differences among FKA, FKB, and FKC arise from variations in the number and positioning of methoxy and hydroxyl functional groups, which influence their physicochemical characteristics and biological activity.

The chemical structures, physicochemical properties, and bioactivities of flavokawains are illustrated in Figure 1.

## 4. Sources of Flavokawain

**Figure 2:**
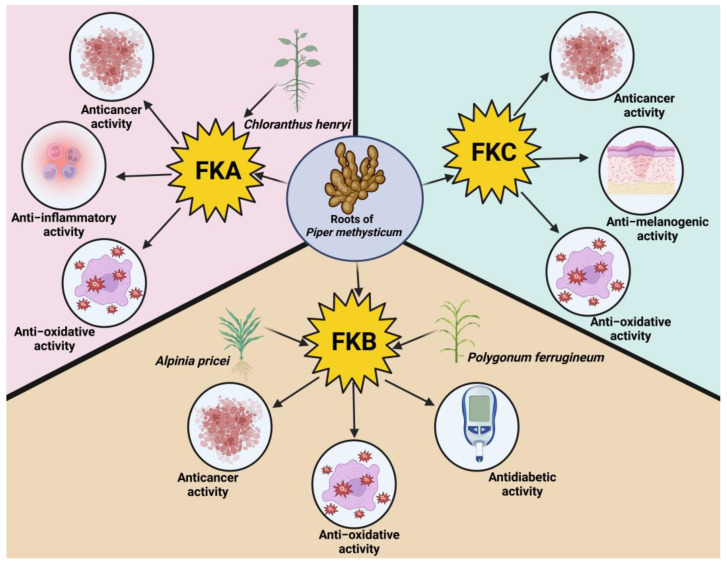
The different plant sources and biological activities of flavokawains. These compounds are predominantly isolated from the roots of *Piper methysticum* (kava plant), with additional sources including *Alpinia pricei, Polygonum ferrugineum*, and *Chloranthus henryi*. The figure integrates phytochemical origin with biological function, highlighting the broad spectrum of pharmacological activities exhibited by flavokawains. Abbreviations: FKA (Flavokawain A), FKB (Flavokawain B), FKC (Flavokawain C).

Flavokawains represent a subclass of naturally occurring chalcones predominantly identified in the kava plant (*Piper methysticum*), a perennial species of the Piperaceae (pepper) family. This plant is indigenous to the South Pacific, including Melanesia, Micronesia, and Polynesia, where it has been traditionally used for its medicinal and ceremonial properties [24,25,26]. FKA, FKB, and FKC are primarily isolated from kava plant roots [23,27]. Among the identified chalcones, FKA is the most abundant, comprising approximately 0.46% of the kava extract, whereas FKB and FKC are present at lower concentrations, at approximately 0.015% and 0.012%, respectively [25]. Another source of FKA, *Chloranthus henryi*, is a herbaceous perennial distributed across the southern regions of China. Traditionally, this herb has been used in Chinese folk medicine to manage several conditions, including pain and bone fractures [28,29]. In addition to *P. methysticum,* FKB has been identified in a rhizomatous herb, *Alpinia pricei* Hayata, indigenous to Taiwan. Traditionally, this plant has been used in herbal medicine to alleviate abdominal bloating and enhance gastric secretion. Furthermore, the leaves of *A. pricei* are commonly employed in the preparation of *zongzi*, a traditional Taiwanese glutinous rice dumpling [30,31]. Notably, *Polygonum ferrugineum Wedd.*, commonly referred to as *caatay guazú*, is a plant native to South America that accumulates the chalcone FKB. In Argentine ethnomedical practices, preparations derived from this plant are employed as natural remedies for their antibacterial, antifungal, wound-healing, and antiseptic effects [32,33]. The plant sources and diverse biological activities of flavokawains are summarized in Figure 2.

## 5. Biological Properties of Flavokawains

Nature offers a wide variety of plant-based resources, which exhibit numerous inherent therapeutic properties that have been widely used in traditional medicine for the management of numerous diseases [34,35]. Similarly, flavokawains have shown substantial promise for treating a range of diseases, attributed to their diverse pharmacological effect and multi-targeted mechanism of action. Some of these diseases include osteoarthritis, oral mucositis, inflammatory bowel disease, idiopathic pulmonary fibrosis, etc. [36,37,38,39]. For example, preclinical evidence suggests that FKA may ameliorate endometriosis by reducing inflammation and angiogenesis while promoting apoptotic cell death [26]. In fibrosis-related contexts, FKA has been shown to interfere with transforming growth factor beta 1 (TGF-β1) signaling in vascular smooth muscle cells by modulating the mothers against decapentaplegic homolog 3 (Smad3) pathway and suppressing reactive oxygen species (ROS) [40]. FKA’s anti-inflammatory activity has been further attributed to inhibition of nuclear factor kappa-light-chain-enhancer of activated B cells (NF-κB) and activator protein 1 (AP-1) activation, along with suppression of c-Jun N-terminal kinase (JNK) and p38 mitogen-activated protein kinase (MAPK) phosphorylation, and downregulation of lipopolysaccharide (LPS)-induced inducible nitric oxide synthase (iNOS) and cyclooxygenase-2 (COX-2) expression in murine macrophages [41]. FKB has been shown to exert significant hypoglycemic activity upon binding to peroxisome proliferator-activated receptor gamma (PPARγ), supporting its potential role in managing type 2 diabetes mellitus. In vitro cytotoxicity assessment in HepG2 and 3T3-L1 cells revealed minimal toxicity at a pharmacologically relevant concentration (5 μM). In vivo evaluation in HFD/STZ-induced and db/db T2DM mouse models demonstrated that FKB did not induce significant weight gain or hepatotoxicity, as indicated by alanine aminotransferase/aspartate aminotransferase (ALT/AST) levels, in contrast to rosiglitazone, thereby supporting its potential as a safer selective PPARγ modulator [42]. FKB was shown to exert a concentration-dependent inhibitory effect on carrageenan-induced mechanical hyperalgesia, likely mediated through the modulation of the nitric oxide (NO)/cyclic guanosine monophosphate (cGMP)/potassium (K^+^) channel signaling pathway [43]. Interestingly, FKB demonstrated significant trypanocidal activity, suggesting its potential activity in the management of trypanosomiasis [33]. In a recent study, FKC was shown to exhibit anti-melanogenic activity when incorporated into nanofibers via electrospinning, a process that significantly enhanced its aqueous solubility and transdermal permeability. The improved formulation led to suppression of melanin synthesis in skin cells by reducing melanin production-associated proteins [23]. The collective findings from the aforementioned studies indicate that flavokawains have enormous potential as therapeutic candidates for managing a variety of diseases. This review delves into the anticancer potential of flavokawains, emphasizing their mechanisms of action and therapeutic efficacy.

A pharmacokinetic evaluation conducted in Sprague Dawley rats following oral administration of FKB at 10 mg/kg demonstrated rapid systemic absorption. The compound achieved a maximum plasma concentration (C_max_) of 265.2 ± 117.6 ng/mL at a time to maximum concentration (T_max_) of approximately 1.00 ± 0.55 h. The systemic exposure, as reflected by the area under the plasma concentration–time curves (AUC_0–24_ and AUC_0–∞_), was 1009.8 ± 601.3 ng h/mL and 1024.2 ± 618.0 ng h/mL, respectively. FKB exhibited a moderate rate of elimination, with an observed terminal half-life (T_1/2_) of 2.76 ± 1.19 h. These results suggest that FKB is efficiently absorbed after oral administration and has a moderate duration of systemic exposure [44].

## 6. Anticancer Potential of Flavokawains

**Figure 3:**
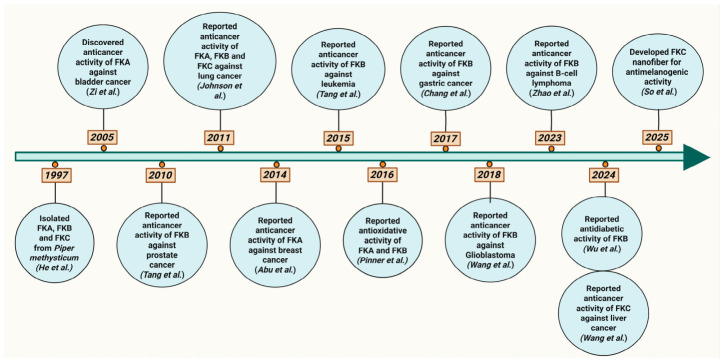
Timeline of major discoveries and pharmacological advancements in flavokawain research. The figure summarizes key experimental discoveries and pharmacological advancements reported between 1997 and 2025, beginning with the initial isolation of flavokawains and subsequently highlighting their progressive evaluation across a wide range of therapeutic applications. Abbreviations: FKA (Flavokawain A), FKB (Flavokawain B), FKC (Flavokawain C) [21,23,42,45,46,47,48,49,50,51,52,53,54].

The anticancer activities of flavokawains have been extensively investigated in in vitro cellular systems and in vivo tumor models. The present review systematically evaluates the accumulating preclinical evidence, highlighting their multi-targeted anti-tumor effects through modulation of oncogenic signaling pathways, induction of apoptosis, suppression of metastasis, and remodeling of the TME. A timeline highlighting key discoveries and pharmacological advancements of flavokawains is presented in Figure 3. A comprehensive summary of the therapeutic effects of flavokawains across various cancer types is provided in Table 2.

### 6.1. B-Cell Lymphoma

**Figure 4:**
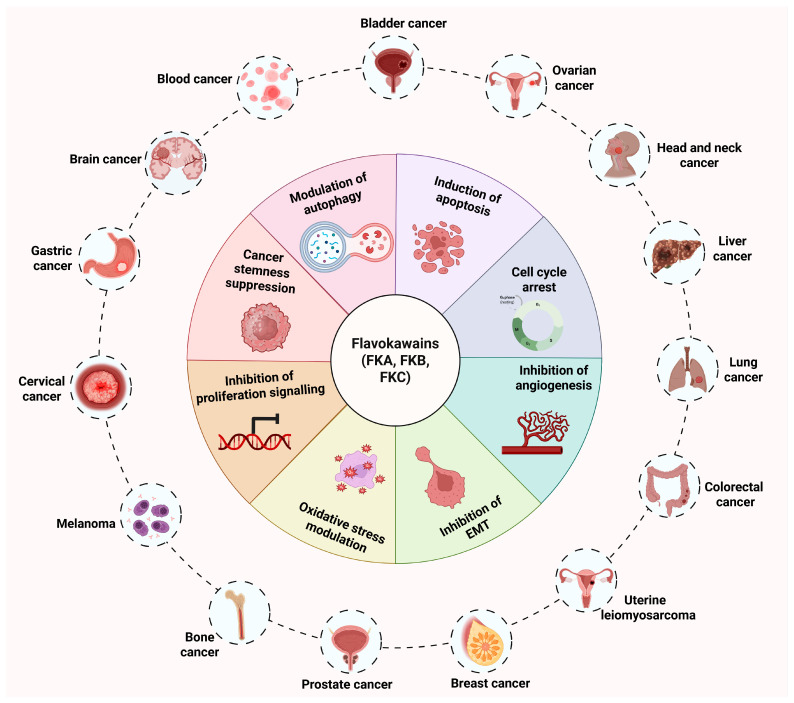
Flavokawains target multiple hallmarks of cancer across diverse malignancies. The figure illustrates the broad-spectrum anticancer activities of flavokawains across a wide range of cancer types, including bladder, ovarian, head and neck, liver, lung, colorectal, uterine leiomyosarcoma, breast, prostate, melanoma, bone, cervical, gastric, brain, and hematological cancers. The central panel highlights the key anticancer mechanisms influenced by flavokawains, including apoptotic activation, cell-cycle blockade, angiogenic restraint, modulation of oxidative stress, attenuation of EMT, disruption of proliferative signaling networks, reduction in cancer stemness, and autophagic remodeling. Abbreviations: FKA (Flavokawain A), FKB (Flavokawain B), FKC (Flavokawain C), EMT (epithelial-mesenchymal transition).

Lymphomas are a heterogeneous group of lymphoproliferative cancers that arise from B cells, T cells, or natural killer cells, and are primarily classified as Hodgkin’s lymphoma (HL) or non-Hodgkin’s lymphoma (NHL) [96,97]. FKB has been reported to exert prominent anti-tumor activity against B-cell lymphoma by interfering with the phosphoinositide 3-kinase (PI3K)/protein kinase B (Akt) signaling pathway [46]. In vitro studies revealed that this compound reduced Akt phosphorylation at Ser^473^, inhibited cell viability in a dose-dependent manner, and triggered apoptosis via downregulation of B-cell lymphoma-extra large (Bcl-xL), activation of caspase-3, and cleavage of poly(ADP-ribose) polymerase (PARP). Also, ABT-199, a selective B-cell lymphoma 2 inhibitor, exhibits synergistic anticancer activity in combination with FKB. The strongest synergy was observed in SUDHL-4 cells, with a combination index below 0.55, compared with Raji and Jeko-1 cells. This combination led to a concentration-dependent reduction in cell viability in B-lymphoma cells. Further, in vivo efficacy was confirmed in an SUDHL-4-derived xenograft model in nude mice, where FKB treatment markedly reduced tumor weight and decreased the number of Ki-67-positive cells [46]. The broad spectrum of anticancer activities of flavokawains across different cancer types is depicted in Figure 4.

### 6.2. Bladder Cancer

Bladder cancer is a highly aggressive neoplasm arising from the urothelium covering the inner lining of the bladder and ranks as the ninth most commonly diagnosed cancer worldwide [5,98]. FKA has attracted interest as a candidate inhibitor of protein arginine methyltransferase 5 (PRMT5), an epigenetic regulatory enzyme linked to poor clinical prognosis in bladder cancer [55]. Liu et al. demonstrated that FKA selectively suppressed the proliferation of PRMT5-expressing bladder cancer cells and promoted apoptosis, accompanied by a marked decrease in the methylation of histone residues H2AR3 and H4R3. In UMUC3 cells derived xenograft model, treatment with FKA led to a significant reduction in tumor size and decreased methylation levels at H2AR3 and H4R3. Importantly, when compared to clinically investigated PRMT5 inhibitors such as EPZ015666 and GSK3326595, FKA exhibited superior anti-tumor activity. Combinatorial treatment with FKA and standard chemotherapeutic agents, including cetuximab, gemcitabine, and cisplatin, resulted in enhanced therapeutic efficacy, suggesting a potential synergistic interaction [55]. A separate study has reported that, in a UPII-SV40T transgenic mouse model of bladder cancer, dietary administration of FKA at a dose of 6 g/kg incorporated into the American Institute of Nutrition 93 maintenance diet (AIN-93M) was associated with prolonged overall survival of mice [56]. Additionally, FKA administration reduced tumor-associated bladder weight. Histopathological analysis revealed that this compound attenuated the progression from carcinoma in situ to more aggressive high-grade papillary and muscle-invasive urothelial carcinoma, suggesting a delay in malignant transformation. Immunohistochemical analysis revealed decreased Ki-67 expression, elevated p27 levels, and increased terminal deoxynucleotidyl transferase dUTP nick end labeling (TUNEL)-positive cells. This pro-apoptotic effect was accompanied by downregulation of anti-apoptotic proteins, including Bcl-2, X-linked inhibitor of apoptosis protein (XIAP), and survivin, and upregulation of the death receptor (DR5) [56]. Another study by Tang Y et al. showed that FKA exhibited differential cell-cycle regulatory effects in bladder cancer cells depending on p53 status [57]. In RT4 cells expressing wild-type p53, FKA triggered G1 phase arrest through upregulation of the cyclin-dependent kinase (CDK) inhibitors p21/WAF1 and p27/Kip1, alongside suppression of S-phase Kinase-associated Protein 2 (SKP2) and CDK2 activity. In T24 cells harboring mutant p53, FKA instead induced G2/M phase arrest accompanied by a time-dependent increase in CDK1 activity, along with a decrease in phosphorylation of Cdc25C at Ser^216^. Moreover, the G2/M checkpoint regulators Myt1 and Wee1 were also downregulated. Oral administration of FKA at 50 mg/kg body weight significantly suppressed tumor growth in RT4-induced xenograft in nude mice, leading to an approximately 64% reduction in tumor volume [57]. Furthermore, FKA reduced cell proliferation in the human bladder cancer cell lines RT4, T24, and EJ by 90–95% [45]. This growth inhibition was accompanied by the induction of apoptosis via activation of caspase-3 and caspase-9 and PARP cleavage, indicating involvement of the intrinsic mitochondrial apoptotic pathway. The anti-apoptotic proteins XIAP and survivin were also downregulated following FKA treatment. Oral administration of FKA at a dosage of 50 mg/kg body weight in nude mice bearing EJ cell-derived xenografts significantly suppressed tumor growth, resulting in a 57% reduction in tumor volume [45]. Interestingly, the combination of FKA with yangonin exhibited a synergistic inhibitory effect on bladder cancer cell proliferation, resulting in a 36–52% greater reduction in cell growth than treatment with either agent alone [58].

Taken together, FKA exhibits significant anti-bladder cancer activity through multiple mechanisms, including inhibition of PRMT5-mediated epigenetic regulation, induction of apoptosis, and modulation of cell-cycle progression. These effects translate into reduced tumor growth, delayed disease progression, and prolonged survival in both xenograft and transgenic bladder cancer models. Moreover, its synergistic effects with conventional chemotherapeutic agents highlight its potential as a promising therapeutic candidate for bladder cancer. However, further safety evaluations and clinical studies are required to validate its efficacy and translational applicability.

### 6.3. Bone Cancer

Bone cancer malignancies are severe, life-threatening conditions that particularly affect children and adolescents worldwide [99]. Flavokawains have gained significant attention for their distinct anticancer properties in the management of various bone-related malignancies. In a pivotal study investigating the therapeutic effect of FKA on osteosarcoma, treatment with FKA resulted in a marked inhibition of cell proliferation [59]. Moreover, FKA significantly suppressed the invasive capabilities of these cells in a concentration-dependent manner. Molecular analyses revealed that FKA downregulated S-phase kinase-associated protein 2 (SKP2) at both transcript and protein levels, along with an increased accumulation of p21 and cleaved caspase-3. FKA also induced G2/M phase arrest and promoted PARP cleavage. In an in vivo model, oral administration of FKA led to a notable reduction in SKP2 expression in lung tissues and a concomitant decrease in p27 expression in metastatic lung nodules, suggesting attenuation of metastatic progression. Additionally, there was a notable increase in TUNEL-positive cells, indicating enhanced apoptosis in metastatic sites [59]. Wang et al. conducted a study using a synovial sarcoma cell model; FKA produced concentration-dependent inhibition of both cellular proliferation and invasive capacity, induced G2/M phase arrest, and triggered apoptosis, as evidenced by the cleavage of PARP and caspase-7. Mechanistic investigations revealed that FKA downregulated SKP2 expression in a dose-dependent manner, suggesting a role in modulating cell-cycle progression and apoptotic sensitivity. Oral administration of FKA at 600 mg/kg, in severe combined immunodeficiency (SCID) mice bearing HSSY-II-induced synovial sarcoma xenografts, significantly reduced tumor volume. Notably, when combined with doxorubicin, FKA exhibited a strong synergistic effect, markedly enhancing cytotoxicity and further reducing synovial sarcoma cell viability beyond the effects observed with either agent alone [60]. Further, FKA dose-dependently inhibited cell proliferation and colony formation in synovial sarcoma cell lines, with induction of apoptosis confirmed by elevated enzymatic activities of caspases-3, -7, -8, and -9, indicating activation of both extrinsic and intrinsic apoptotic pathways. Pro-apoptotic regulators such as DR5, Bim, and Puma were elevated, while survivin expression was reduced. A shift in the balance of Bcl-2 family proteins was also observed, with an upregulation of BCL2-associated X protein (Bax) and a downregulation of Bcl-2, further contributing to the pro-apoptotic effect of FKA [61].

Collectively, these findings demonstrate that FKA possesses significant anti-tumor activity against osteosarcoma and synovial sarcoma by inhibiting cell proliferation and invasion, inducing G2/M cell-cycle arrest, promoting apoptosis, and suppressing SKP2-mediated oncogenic signaling. The observed reduction in tumor growth and metastasis, along with its synergistic effects with doxorubicin, highlights the potential of FKA as a promising therapeutic candidate for bone-related malignancies. However, further clinical studies are required to establish its safety and efficacy in humans.

### 6.4. Breast Cancer

Breast cancer is a highly aggressive malignancy and ranks as the second most frequently diagnosed cancer worldwide, with strong propensity to metastasize to distant organs including bone, liver, lung and brain [5,100,101]. Advancements in synthetic derivatives of FKB have unveiled their potency in breast cancer treatments. For instance, a series of twenty-three FKB derivatives (compounds **1**–**23**) was assessed for anticancer activity in breast cancer cell lines. Among these, compounds FKB-13, FKB-15, and FKB-16 demonstrated markedly enhanced cytotoxic effects relative to the other compounds in the series, warranting further development [62]. In human epidermal growth factor receptor 2 (HER2)-overexpressing breast cancer, FKA has demonstrated an inhibitory effect on cell proliferation, significantly suppressing clonogenic capacity by up to 80% and inducing G2/M phase arrest [63]. Mechanistically, FKA activated Cdc2 kinase activity while concurrently decreasing its phosphorylation at Tyr^15^, which correlated with reduced expression of its upstream inhibitory regulators, Wee1 and Myt1. FKA also promoted apoptosis in these cells by upregulating Bim and Bax and suppressing survivin, XIAP, Bcl-2, and Bcl-xL. Notably, co-treatment with Herceptin yielded a synergistic reduction in cell viability, highlighting the potential of FKA as an effective adjunct in targeting HER2-driven breast cancer [63]. Further supporting these findings, FLS, a synthetic derivative of flavokawain, exhibited significant antiproliferative effects in breast cancer cells by triggering apoptosis and reducing cell viability, and was associated with G2/M cell-cycle arrest and a progressive increase in the expression of p53 and Bax, alongside decreased Bcl-2 [64]. Additionally, FLS treatment led to elevated caspase-9 activity and enhanced time-dependent cytosolic release of cytochrome c, implicating activation of the intrinsic mitochondrial apoptotic pathway in its mechanism of action [64]. Similarly, a synthesized form of FKB also suppressed breast cancer cell proliferation in a dose-dependent manner, accompanied by induction of apoptosis and G2/M phase arrest of the cell cycle. Under ex vivo conditions, FKB significantly reduced both the invasive and migratory capabilities of breast cancer cells in a concentration-dependent manner and impaired angiogenic activity, further supporting its potential as a multi-targeted therapeutic agent against breast cancer progression [65]. Another study revealed that FKA demonstrated a dose-dependent inhibition of breast cancer cell proliferation, achieving approximately 50% growth suppression at concentrations below 50 µM [21]. The compound exhibited greater potency in MDA-MB-231 cells (IC_50_ = 17.49 µM) than in MCF-7 cells (IC_50_ = 25.13 µM). Mechanistic investigations revealed that FKA induced G2/M arrest, promoted apoptosis through activation of caspase-8 and -9, upregulation of pro-apoptotic markers such as Bax and cytochrome c, and downregulation of the mitotic regulators Polo-like kinase 1 (PLK1) and Forkhead box protein M1 (FOXM1). In functional assays, FKA reduced the invasive and migratory behavior of cancer cells in a concentration-dependent manner. Additionally, its anti-angiogenic properties were confirmed using the rat aortic ring model, in which FKA effectively inhibited vessel outgrowth from aortic fragments, supporting its potential as a multi-targeted agent in breast cancer therapy [21].

Flavokawains have demonstrated broad-spectrum preclinical anti-tumor activity against breast cancer through diverse mechanisms, including inhibition of cell proliferation, induction of apoptosis via intrinsic and extrinsic pathways, G2/M cell-cycle arrest, suppression of invasion and migration, and inhibition of angiogenesis. The synergistic efficacy of FKA with Herceptin in HER2-overexpressing breast cancer, along with the enhanced cytotoxic potential of synthetic derivatives such as FKB-13, FKB-15, FKB-16, and FLS, further supports the translational promise of this class of compounds. Nonetheless, advancement toward clinical application necessitates comprehensive in vivo safety and efficacy validation, as well as well-structured clinical trials to firmly establish the therapeutic utility of flavokawains in breast cancer management.

### 6.5. Cervical Cancer

Cervical cancer is a leading female malignancy that contributes significantly to the global health burden, ranking as the fourth most commonly diagnosed cancer among females worldwide [5,102]. An investigation into the effects of FKB on HeLa cells revealed cytotoxic activity, with an IC_50_ of 17.5 µM. FKB treatment also resulted in G2/M phase arrest and induced apoptosis, indicating its potential to disrupt cellular proliferation and promote programmed cell death [66]. Furthermore, a notable upregulation of intracellular antioxidant markers, including superoxide dismutase (SOD) and glutathione (GSH), was observed in FKB-treated cells, suggesting enhanced antioxidant defense. When combined with hydrogen peroxide (H_2_O_2_), FKB markedly attenuated reactive oxygen species (ROS) levels, implying its role in modulating oxidative stress within the cellular environment [66]. FKB has exhibited promising preclinical anticancer activity in cervical cancer, as evidenced by its cytotoxic effects, induction of G2/M arrest and apoptosis, and modulation of the cellular oxidative stress environment through upregulation of the antioxidant markers SOD and GSH.

These findings show the multifaceted mechanistic potential of FKB as a therapeutic candidate in cervical cancer management. However, the current evidence remains limited to a single in vitro study, and extensive preclinical investigations encompassing in vivo models, followed by rigorous clinical evaluation, are imperative to comprehensively establish its therapeutic efficacy in this malignancy.

### 6.6. Cholangiocarcinoma

Cholangiocarcinoma is a highly aggressive malignancy arising from the epithelial cells of the bile ducts or liver [103,104]. To improve therapeutic efficacy, combinatorial approaches are being evaluated. For instance, a combination treatment of FKB and cisplatin produced a synergistic reduction in cell viability and enhanced apoptosis in cholangiocarcinoma cells, with effects increasing in a dose-dependent manner relative to monotherapy with either agent [22]. Mechanistically, this enhanced apoptotic response was associated with suppression of the Akt signaling pathway. Additionally, co-treatment led to a marked elevation in cleaved PARP expression, indicating increased apoptotic activity compared to single-agent treatments. In vivo co-treatment of FKB with cisplatin or gemcitabine markedly inhibited tumor growth in SNU-478 xenograft-bearing nude mice, further supporting the therapeutic potential of FKB as a sensitizing agent to cisplatin or gemcitabine in cholangiocarcinoma [22]. Despite these promising preclinical findings, further investigations are required to comprehensively evaluate the therapeutic efficacy of all three flavokawains and to determine their potential integration into standard treatment strategies for cholangiocarcinoma.

### 6.7. Colorectal Cancer

Globally, colorectal cancer is diagnosed in approximately two million individuals each year, ranking as the third most commonly diagnosed malignancy and second most common cause of death worldwide, with an estimated 60% rise worldwide by 2030 [105,106]. In a recent study, FKA exhibited concentration-dependent cytotoxicity in human colorectal cancer cells, with a marked reduction in cell viability at elevated concentrations [67]. Supporting these findings, another study [71] demonstrated that treatment with FKC significantly reduced cell viability and induced G2/M phase arrest through upregulation of p21 and p27. Apoptotic induction was confirmed by activation of caspases-3, -8, and -9, indicating involvement of both intrinsic and extrinsic pathways. Additionally, FKC suppressed the expression of IAPs, including XIAP, c-IAP1, and c-IAP2. A dose-dependent increase in intracellular ROS levels was accompanied by a decrease in SOD activity, suggesting that oxidative stress may contribute to FKC-induced cell death [71]. Proteomic profiling following FKC treatment revealed significant modulation of specific proteins. Notably, 17 proteins were upregulated, including molecular chaperones and stress-response proteins such as Hspa8, Hsp70-Hom, Hsp27, Hsp70-1/2, and Hsp86, as well as cytoskeletal and metabolic regulators such as TCP-1-eta, CK-18, Tubulin beta-2 chain, and Gamma actin. Additional upregulated proteins included P4HB, heme oxygenase-1 gene (HMOX1), FKBP4, HR23B, SFPQ, PARK7, GLOD4, and GLRX3, suggesting an activation of stress adaptation and redox homeostasis pathways. Conversely, 18 proteins were downregulated in response to FKC, including proteins involved in cytoskeletal function (MLC-3), metabolism (GAMT, PGAM1), detoxification (GSTO1, COMT), and protein synthesis or degradation pathways (DCK, eIF-5A1, EF-2, eIF-3I, Skp1, CBX3, TCEB1, RanBP1, hTom22, and ATP5H) [70]. Moreover, in a xenograft model using HCT-116 colorectal cancer cells in nude mice, administration of FKC at doses of 1 mg/kg and 3 mg/kg produced tumor growth inhibition of approximately 18.7–24.0% and 23.4–52.2%, respectively [68]. Importantly, FKC treatment was well tolerated, without notable changes in body weight or systemic toxicity indicators. Histological analysis of tumor tissues revealed an increase in TUNEL-positive cells and elevated levels of cleaved caspase-3, indicative of apoptosis induction. Concurrently, a reduction in Ki-67-positive cells was observed, reflecting suppression of cell proliferation [68]. In another study, FKB was assessed for its anticancer potential in human colorectal cancer cells, where it demonstrated a dose-dependent inhibition of cell growth. This antiproliferative effect was accompanied by a notable reduction in proliferating cell nuclear antigen (PCNA) expression. Mechanistic analysis revealed activation of caspase-3, indicating the induction of apoptosis. Additionally, FKB treatment led to cell-cycle arrest at the G2/M phase, suggesting that disruption of cell-cycle progression contributes to its cytotoxic activity [69]. Furthermore, FKB was shown to exert dose-dependent antiproliferative activity in vitro, significantly suppressing clonogenic capacity and inducing G2/M phase arrest [72]. Apoptosis was triggered via a mitochondria-dependent intrinsic pathway, leading to caspase activation, as indicated by cytochrome c release. Additionally, FKB treatment upregulated growth arrest and DNA damage-inducible protein 153 (GADD153), elevated intracellular ROS levels, and promoted autophagy, suggesting its multifaceted role in disrupting cancer cell survival [72]. In a recent study, researchers investigated the anti-CRC effects of FKA using an AOM/DSS-induced colorectal cancer model in mice [73]. FKA treatment produced a significant, dose-dependent reduction in the number of colorectal tumors and polyp size, as confirmed by hematoxylin and eosin (H&E) histopathology. Employing integrated 16S rRNA sequencing and untargeted metabolomics, the study further elucidated the underlying mechanisms. FKA restored gut microbiota diversity, evidenced by improved α-diversity indices, including Chao1, Shannon, and Faith’s PD. Further, PICRUSt2 functional prediction revealed that FKA significantly downregulated the LPS biosynthesis superpathway, a key pro-inflammatory endotoxin route, thereby attenuating the inflammatory-to-carcinogenic transition in the colon. Untargeted metabolomics of colonic tissue further demonstrated that FKA inversely modulated lipid and arachidonic acid metabolic pathways, thereby suppressing the generation of pro-inflammatory eicosanoids, including prostaglandins and leukotrienes [73].

Collectively, the available evidence demonstrates that flavokawains, particularly FKA, FKB, and FKC, exhibit potent anticancer activity against colorectal cancer through multiple mechanisms, including inhibition of cell proliferation, induction of G2/M cell-cycle arrest, promotion of apoptosis, generation of oxidative stress, and modulation of key survival pathways. In addition, FKA has shown chemopreventive potential by suppressing colorectal tumor formation, restoring gut microbiota homeostasis, and attenuating inflammation-associated carcinogenic pathways. These findings highlight the promise of flavokawains as potential therapeutic and preventive agents for colorectal cancer; however, further clinical studies are required to validate their safety and efficacy in humans.

### 6.8. Gastric Cancer

The incidence of gastric cancer increases markedly with age, particularly between 55 and 80 years, and is approximately threefold higher in men compared to women [107]. FKB has been evaluated in multiple gastric cancer cell lines, where it consistently inhibited cell viability and proliferation and induced apoptosis and autophagy [49,74,75]. Treatment with FKB promoted cytochrome-c release and activated caspase-9, implicating the mitochondrial apoptotic pathway. Concurrently, increased expression of Fas and FasL, along with activation of caspase-8, indicated engagement of the extrinsic apoptotic pathway [74]. FKB also induced G2/M phase arrest by reducing Cyclin A, Cyclin B1, CDK1, Cdc25C, and Cdc2 [49,75]. Interestingly, treatment reduced the levels of both Bcl-2 and Bax [49]. In addition, FKB modulated oncogenic signaling cascades, including PI3K/Akt/mammalian target of rapamycin (mTOR), ROS-JNK, and TGF-β [49,74,75]. In vivo, administration of FKB significantly reduced tumor volume and significantly upregulated TSPAN12, TGF-β1, and SMAD4 proteins in AGS- and SGC-7901-derived xenograft models [49,74,75]. Furthermore, the combination of FKB with doxorubicin enhanced cytotoxicity and autophagy in gastric cancer cells in vitro and produced marked inhibition of tumor growth in vivo. Notably, the synergistic effect between FKB and doxorubicin was further confirmed by a combination index < 1 [74].

These findings warrant further investigation to elucidate the effects of FKB on key processes, including cell invasion, migration, and metastasis, in gastric cancer cells. In addition, evaluating its efficacy across a broader panel of gastric cancer cell lines is necessary to validate its clinical potential. Moreover, the anticancer effects of related flavokawains, including FKA and FKC, should also be explored in gastric cancer models.

### 6.9. Head and Neck Cancers

Oral squamous cell carcinoma (OSCC) is the predominant form of oral cancer and is mostly diagnosed at an advanced stage [108,109]. At relatively low concentrations of 10 μg/mL for FKA and 2.5 μg/mL for FKB, both compounds significantly suppressed cell growth in OSCC cells. In addition to their antiproliferative effects, both flavokawains markedly diminished the migratory and invasive potential of OSCC cells [76]. In addition, FKB inhibited the growth of oral carcinoma cells by inducing apoptosis and causing G2/M cell-cycle arrest [77]. This was associated with reduced expression of cyclin A, cyclin B, Cdc2, and Cdc25C. FKB also promoted the proteolytic activation of procaspase-9 and -3, decreased Bcl-2 levels, and increased Bax expression. Additionally, FKB suppressed PI3K/Akt phosphorylation and p38 MAPK signaling, while inducing a time-dependent upregulation of JNK1/2 and extracellular signal-regulated kinase (ERK1/2), indicating its involvement in both apoptotic and stress-related signaling pathways [77]. Further, under in vitro conditions, FKB inhibited cell proliferation by approximately 85% in oral adenoid cystic carcinoma cells, primarily by inducing G2/M arrest. Additionally, FKB triggered apoptosis and dose-dependently upregulated mRNA expression of the pro-apoptotic markers Bim, Bak, and Bax, as well as Bcl-2, suggesting involvement of mitochondrial apoptotic pathways [78].

Over recent decades, the incidence of nasopharyngeal carcinoma, a highly aggressive malignancy of the head and neck, has shown a marked increase [110]. Treatment with FKC notably inhibited cell proliferation and triggered apoptosis in nasopharyngeal carcinoma cells, primarily by targeting heat shock protein 90 beta family member 1 (HSP90B1) and reducing the expression of the angiogenic factors angiopoietin-1 (Ang-1) and vascular endothelial growth factor (VEGF) [79]. Additionally, FKC suppressed the phosphorylation of key components within the epidermal growth factor receptor EGFR/PI3K/Akt/mTOR signaling cascade, contributing to its anti-tumor effects. In vivo, FKC administration led to a substantial decrease in both tumor volume and weight. This was associated with downregulation of HSP90B1, glucose transporter 1 (GLUT1), hexokinase 2 (HK2), Ang-1, and VEGF, further supporting the inhibition of the EGFR/PI3K/Akt/mTOR axis and related metabolic and angiogenic processes [79].

Collectively, these studies demonstrate that flavokawains exert potent anti-tumor effects against head and neck malignancies, including oral squamous cell carcinoma, oral adenoid cystic carcinoma, and nasopharyngeal carcinoma. Their anticancer activity is mediated through the suppression of cell proliferation, migration, invasion, angiogenesis, and tumor growth, alongside the induction of apoptosis and cell-cycle arrest. Mechanistically, flavokawains modulate multiple oncogenic signaling pathways, including PI3K/Akt, MAPK, EGFR/PI3K/Akt/mTOR, and mitochondrial apoptotic pathways, highlighting their potential as promising multi-target therapeutic agents for the management of head and neck cancers.

### 6.10. Liver Cancer

Liver cancer accounted for approximately 905,677 new cases and 830,180 fatalities globally, with both incidence and mortality rates significantly higher in males compared to females [111]. The effect of flavokawains was investigated in liver cancer. For example, FKA treatment suppressed the viability, invasive capacity, migration, and vasculogenic mimicry of hepatocellular carcinoma (HCC) cells, accompanied by reduced expression of VE-cadherin, vimentin, and Snail1, and increased E-cadherin levels, indicative of epithelial–mesenchymal transition (EMT) inhibition [29]. This effect was associated with decreased phosphorylation of PI3K and Akt, and with downregulation of key EMT-associated transcription factors, including HIF-1α, NF-κB, and Twist1. In a HepG2 xenograft model, FKA administration suppressed tumor growth and metastasis, elevated E-cadherin expression, and suppressed Twist1, VE-cadherin, vimentin, and p-Akt levels, suggesting that FKA hinders EMT progression by targeting the PI3K/Akt/Twist1 signaling axis [29]. Another study compared the cytotoxicity of FKA and FKB in HepG2 cells and found that FKA exhibited less cytotoxicity than FKB [50]. Both compounds induced a concentration-dependent increase in nuclear factor erythroid 2-related factor 2 (Nrf2) and HSF1 expression, as well as elevated mRNA levels of antioxidant and stress-response genes, including HMOX1 and GCLC. Additionally, dose-dependent upregulation of HSPA1A and DNAJA4 was observed. Protein levels of HO-1 and Hsp70-1 were also increased following treatment with FKA and FKB. However, both compounds were shown to enhance intracellular GSH levels and confer protection against H_2_O_2_-induced oxidative cell death, suggesting a role in cellular defense through activation of antioxidant and heat shock responses in these cells [50]. Therefore, more studies are required to validate these findings as both compounds induce resistance in cancer cells, which is not good for cancer treatment. Expanding the investigation to FKC, a recent study reported that treatment of liver cancer cells with FKC resulted in inhibition of cell proliferation and clonogenic capacity, along with induction of apoptosis, as evidenced by decreased Bcl-2 and elevated Bax protein levels [48]. FKC also promoted DNA damage, indicated by increased γ-H2AX expression. Additionally, it impaired cell adhesion and significantly suppressed migration, which correlated with reduced phosphorylation of focal adhesion kinase (FAK), PI3K, and Akt, suggesting disruption of key signaling pathways involved in cell motility and survival. In vivo, FKC administration led to reduced tumor growth, lower Ki-67 expression, and elevated γ-H2AX levels, indicating reduced proliferation and increased DNA damage in a Huh-7 xenograft mouse model [48].

Collectively, these findings suggest the promising anticancer potential of flavokawains in liver cancer; however, further studies are warranted to fully elucidate their mechanisms of action, address concerns about chemoresistance, and validate their therapeutic efficacy in clinical settings.

### 6.11. Lung Cancer

Lung cancer is the most commonly diagnosed malignancy globally, with an estimated 2.5 million new cases annually [112]. FKA induced a concentration-dependent inhibition of cell viability in lung cancer cells by inducing apoptosis as confirmed by PARP cleavage, while exhibiting no toxicity toward normal human hepatic epithelial THLE-3 cells. Additionally, FKA markedly suppressed P-glycoprotein, Akt, and p-Akt (Ser^473^), suggesting its potential role in overcoming drug resistance through modulation of the PI3K/Akt signaling pathway [80]. Additionally, in a murine model of tumorigenesis induced by 4-(Methylnitrosamino)-1-(3-pyridyl)-1-butanone (NNK) and benzo[a]pyrene (BaP), treatment with FKA, FKB, and FKC each reduced tumor multiplicity by 27%, 34%, and 23%, respectively, suggesting a differential yet significant chemopreventive effect of each flavokawain [51]. Furthermore, FKB markedly reduced cell viability and clonogenic potential in lung cancer cells, while promoting apoptosis, as indicated by increased TUNEL-positive cells, activation of caspase-3 and -9, and subsequent PARP cleavage. Additionally, FKB induced ROS generation, contributing to oxidative stress-mediated cell death. An increase in microtubule-associated protein 1A/1B-light chain 3-II (LC3-II) expression was observed, suggesting the induction of autophagy. Concurrently, FKB treatment led to reductions in Bcl-2 and Bax protein levels, implicating disruption of mitochondrial integrity [82]. In another study, Chalcone-24, a synthetic flavokawain, was found to suppress both cell viability and NF-κB activation in lung cancer cells. At lower concentrations, treatment with Chalcone-24 elevated ERK1/2 and JNK phosphorylation, accompanied by enhanced caspase activity [81].

Collectively, these findings indicate that flavokawains possess significant anti-lung cancer activity by inhibiting tumor cell growth, inducing apoptosis and autophagy, suppressing oncogenic signaling pathways, and reducing tumor burden. However, further pharmacokinetic, toxicity, and clinical studies are required to validate their therapeutic potential.

### 6.12. Leukemia

An increase in white blood cell count in the blood and bone marrow is characteristic of this hematological malignancy [113]. Leukemia represents a notable global health burden, accounting for about 2.5% and 3.1% of all global cancer cases and mortality, respectively [114]. In a recent study, FKA was shown to induce a concentration-dependent reduction in the viability of acute myeloid leukemia (AML) cells and trigger G1 phase arrest through the downregulation of CCND1, CCNE1, CCNE2, CDK2, CDK4, CDK6, and CDT1, along with upregulation of the CDK inhibitor CDKN1B [83]. In ex vivo experiments using primary leukemic cells isolated from AML patients, FKA similarly reduced cell viability in a dose-dependent manner, with IC_50_ values ranging from 3.86 to 9.45 μg/mL, reinforcing its therapeutic potential in AML [83]. Another study showed that FKB markedly inhibited proliferation and induced apoptosis in acute lymphoblastic leukemia (ALL) cells in vitro, in a dose-dependent manner, by enhancing caspase-3 activation and PARP cleavage. FKB treatment also led to a concentration-dependent upregulation of key pro-apoptotic regulators, including p53, Bax, and Puma. In vivo, FKB administration resulted in a notable reduction in leukocytes, white blood cell counts, and a decrease in splenomegaly, indicating systemic anti-leukemic activity. Ex vivo analysis of patient-derived B-ALL and T-ALL confirmed these findings, showing marked suppression of cell proliferation and elevated levels of p53, Bax, and Puma [52]. Further investigation demonstrated that FKB enhanced daunorubicin-induced reduction in cell viability, demonstrating an additive cytotoxic effect. Interestingly, rather than suppressing NF-κB activity, the combination treatment activated NF-κB, suggesting a complex interaction between FKB and daunorubicin [84].

Taken together, flavokawains exhibit potent anti-leukemic effects through the induction of cell-cycle arrest, activation of apoptotic pathways, and enhancement of chemotherapeutic efficacy, supporting their potential as novel therapeutic agents against leukemia. Nevertheless, further pharmacokinetic, toxicological, and clinical investigations are needed to facilitate their translation into leukemia therapy.

### 6.13. Melanoma

Melanoma, particularly in its advanced stages, presents a significant clinical challenge and is often associated with a poor prognosis [115]. In addition, synthetic flavokawains A (FLA) and B (FLB) demonstrated potent cytotoxic effects, reducing cell viability by approximately 85% and 80%, respectively. Both compounds induced a dose-dependent decrease in intracellular melatonin levels, indicating suppression of melatonin biosynthesis. This was further supported by reduced expression of key melanogenic markers, including tyrosinase (Tyr), tyrosinase-related proteins 1 and 2 (Trp-1, Trp-2), and microphthalmia-associated transcription factor (Mitf). In a zebrafish toxicity model, both FLA and FLB exhibited high survival rates, indicating low systemic toxicity while still effectively reducing melatonin content [85].

### 6.14. Neuro-Oncological Malignancies

Glioblastoma (GBM) is a highly aggressive and uniformly fatal primary brain tumor, classified as a grade IV astrocytoma, and accounts for over 60% of all brain tumor cases [116,117]. To examine newer treatment modalities, FKB was studied in vitro in glioblastoma cells, where it produced a concentration-dependent reduction in cell viability [53]. This compound further induced G2/M arrest and promoted cellular senescence, as evidenced by the accumulation of senescence-associated β-galactosidase (SA-β-gal)-positive cells. Markers of autophagy, including increased MAP1LC3B-II and decreased SQSTM1 expression, indicated activation of the autophagic pathway. This autophagy appeared to be dependent on endoplasmic reticulum (ER) stress, as evidenced by the upregulation of ER stress-associated proteins, including HSPA5, phosphorylated EIF2AK3 and EIF2A, ATF4, and DDIT3. Additionally, FKB suppressed the phosphorylation of Akt, mTOR, and RPS6KB1, thereby inhibiting the Akt-mTOR-RPS6KB1 signaling axis and further implicating this pathway in autophagy induction by this compound. Moreover, in vivo administration of FKB at 50 mg/kg significantly reduced tumor growth in a U251 glioblastoma xenograft model in nude mice, indicating its potential anti-tumor efficacy [53]. However, effective glioblastoma treatment requires penetration of the blood–brain barrier (BBB), and there is currently no compelling evidence that FKB can adequately traverse it. Therefore, further investigations are required to evaluate its BBB permeability and elucidate potential strategies or delivery mechanisms to facilitate its transport across the BBB, thereby enhancing its suitability as a therapeutic candidate for glioblastoma treatment.

Despite the development of various therapeutic approaches, neuroblastoma remains associated with a high risk of relapse and disease-related mortality [118]. FKA exerted a dose-dependent suppression of cell proliferation and clonogenic potential in neuroblastoma cells by inducing G1-phase cell-cycle arrest and promoting apoptosis. In addition to its antiproliferative effects, FKA significantly impaired the cells’ invasive and migratory capabilities and downregulated EMT markers, including N-cadherin and Snail. Furthermore, FKA reduced VE-cadherin expression in a dose-dependent manner, indicating its capacity to inhibit angiogenesis and suppress tumor progression [86].

Collectively, these findings demonstrate that flavokawains exert promising anti-tumor effects against neuro-oncological malignancies by suppressing cell proliferation, inducing cell-cycle arrest, apoptosis, autophagy, and senescence, and inhibiting invasion, migration, angiogenesis, and EMT. Mechanistically, these effects are mediated through modulation of ER stress-associated pathways, inhibition of Akt/mTOR signaling, and regulation of EMT-related markers. However, further studies are required to elucidate their pharmacokinetic properties, validate their efficacy in clinically relevant preclinical models, and, particularly for glioblastoma, determine their ability to penetrate the BBB and achieve therapeutically effective concentrations within the central nervous system.

### 6.15. Ovarian Cancer

Ovarian cancer is a heterogeneous malignancy that frequently affects women under the age of 40 and ranks as the eighth most common cancer among females worldwide [119]. The combinatorial administration of FKA-A and PTX-A nanoparticles markedly reduced proliferation and clonogenic capacity in ovarian cancer cell lines. This treatment also significantly impaired cellular migratory ability, as evidenced by elevated E-cadherin and reduced vimentin expression, indicating suppression of EMT. Furthermore, in an A2780 xenograft mouse model, the combined therapy substantially inhibited tumor growth [87]. Another study reported that FKC led to a notable reduction in ovarian cancer cell viability [88]. However, further preclinical and clinical investigations are warranted to confirm the therapeutic potential of flavokawains in ovarian cancer and facilitate their clinical translation.

### 6.16. Prostate Cancer

Prostate cancer is a major malignancy that poses a significant health concern for the male population [120]. FKA treatment produced a significant reduction in both the size and number of prostaspheres derived from DU145 and 22Rv1 cancer stem cells (CSCs), accompanied by downregulation of stemness-associated markers Oct4, Sox2, and Nanog. In vitro, FKA dose-dependently suppressed c-Myc expression in prostaspheres from both cell lines. In an in vivo model, dietary administration of FKA resulted in a 48% reduction in tumor volume, a 64% decrease in Ki-67-positive proliferating cells, and a marked decline in CD44-positive CSCs. Additionally, FKA inhibited Ubc12 neddylation and reduced c-Myc levels in xenograft tumor tissues [89]. In addition, FKA was shown to suppress proliferation in prostate cancer cells by inducing apoptosis and causing cell-cycle arrest at the G2/M phase, an effect associated with decreased survivin expression. Additionally, FKA treatment led to a dose-dependent reduction in intracellular GSH levels and glutathione synthetase (GSS) activity, resulting in a marked increase in ROS accumulation [90]. Also, FKB inhibited the neddylation of Cullin1 and Ubc12 by directly binding to the NEDD8-activating enzyme (NAE) regulatory subunit APP-BP1, thereby impairing SCF^SKP2^ complex function. This results in enhanced SKP2 ubiquitination and proteasomal degradation without affecting proteasome activity. This promoted SKP2 ubiquitination and proteasomal degradation without affecting proteasome activity, leading to reduced SKP2 expression and increased p27/Kip1 levels in prostate cancer cells [91]. Furthermore, FKB induced a ~90% reduction in prostate cancer cell viability at 17.6 µM, primarily via activation of caspases-3, -8, and -9, indicating apoptosis via intrinsic and extrinsic pathways. Upregulation of DR5 enhanced TRAIL-mediated apoptosis, while increased Bim and Puma and decreased XIAP and survivin further supported pro-apoptotic effects. In DU145 xenograft mice, FKB (50 mg/kg/day) reduced tumor volume by ~67% and increased Bim expression in tumor tissues [54]. Moreover, FKA significantly inhibited prostate cancer cell growth by approximately 93% in Rb-deficient DU145 cells and reduced the viability of Rb-deficient MEFs by 92%. The increased sensitivity of SKP2-overexpressing cells to FKA suggests that SKP2 may be a potential target of FKA in pRb-deficient prostate cancers. FKA treatment also decreased NEDDylation of Cullin1 and Ubc12, indicating disruption of protein degradation pathways. In a transgenic adenocarcinoma of mouse prostate (TRAMP) mouse model, dietary FKA suppressed high-grade prostatic intraepithelial neoplasia (HG-PIN) lesions by 69% and prostate adenocarcinomas by 43%, resulting in an overall 73% reduction in tumors. This was accompanied by decreased proliferation, enhanced apoptosis, and absence of metastasis [92]. In another study, FKB treatment led to a marked reduction in androgen receptor (AR) expression and prostate-specific antigen (PSA) protein levels. When combined with other kavalactones, FKB suppressed prostate cancer cell growth by over 70%. In patient-derived xenografts in SCID mice, FKB inhibited tumor growth by approximately 77.3% and significantly reduced serum PSA levels by the end of treatment [93].

Collectively, these studies demonstrate that flavokawains exert potent anti-prostate cancer activity by suppressing tumor cell proliferation, cancer stemness, androgen receptor signaling, and tumor progression while promoting cell-cycle arrest, oxidative stress, and apoptosis. Mechanistically, flavokawains target multiple oncogenic pathways, including NEDDylation, Skp2-mediated signaling, c-Myc regulation, and AR/PSA signaling, and exhibit efficacy in both castration-sensitive and castration-resistant prostate cancer models. Furthermore, their significant tumor-suppressive effects in xenograft and transgenic mouse models underscore their therapeutic potential.

### 6.17. Squamous Carcinoma

In line with findings observed in other cancer types, flavokawain compounds have also been investigated in squamous carcinoma models, demonstrating significant anticancer activity. FKB treatment reduced cell viability and induced G2/M phase arrest in KB and HGF cells, accompanied by increased ROS production and mitochondrial dysfunction [94]. This was associated with cytochrome c release, activation of caspase-3 and -9, and upregulation of Fas and FasL, indicating activation of both intrinsic and extrinsic apoptotic pathways. FKB also downregulated Bcl-2 and upregulated Bax. Cell-cycle regulators, including cyclin A, cyclin B1, Cdc2, and Cdc25C, were suppressed, while p21/WAF1, Wee1, and p53 were upregulated. Additionally, FKB reduced the expression of the metastasis-associated proteins matrix metalloproteinase (MMP-9) and urokinase-type plasminogen activator (u-PA) and increased the expression of their inhibitors, tissue inhibitor of metalloproteinase-1 (TIMP-1) and plasminogen activator inhibitor-1 (PAI-1), in a dose-dependent manner. In vivo, FKB administration (0.75 mg/kg) significantly reduced tumor volume, suppressed angiogenesis, and increased TUNEL-positive cells, indicating enhanced apoptosis and reduced tumor cell proliferation in KB xenograft mice model [94].

### 6.18. Uterine Leiomyosarcoma

Uterine leiomyosarcoma is a highly aggressive malignancy characterized by frequent recurrence, metastatic potential, and poor clinical outcomes [121]. FKB markedly inhibited uterine leiomyosarcoma cell growth by approximately 80%, induced G2/M cell-cycle arrest, and triggered apoptosis. This was associated with upregulation of pro-apoptotic proteins Bim, Puma, and DR5, along with downregulation of IAP and survivin. Notably, FKB exhibited a synergistic effect when combined with gemcitabine or docetaxel, resulting in enhanced suppression of cell proliferation [95].

**Figure 5:**
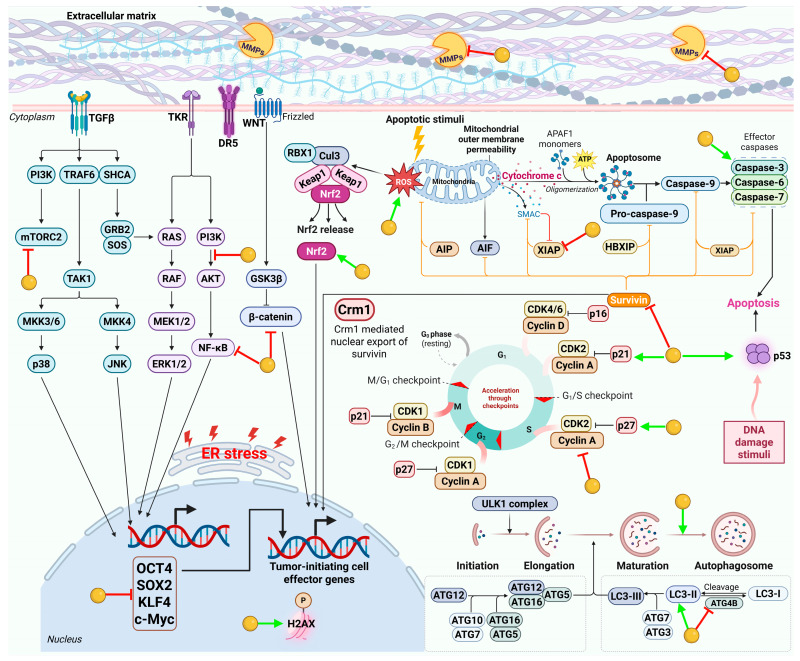
Molecular signaling pathways targeted by flavokawains in cancer. This figure illustrates the multi-targeted anticancer mechanisms of flavokawains. Flavokawains inhibit key oncogenic signaling pathways, including PI3K/Akt/mTOR, NF-κB, MAPK, and Wnt/β-catenin, thereby suppressing proliferation and tumor-initiating gene expression. It reduces metastasis by inhibiting MMPs. Flavokawains induce oxidative stress and endoplasmic reticulum stress, leading to mitochondrial dysfunction, cytochrome c release, caspase activation, and apoptosis, as well as downregulation of XIAP and survivin. It also promotes DNA damage (γ-H2AX), activates p53, and causes cell-cycle arrest via modulation of cyclins, CDKs, p21, and p27. Additionally, FKB triggers autophagy by regulating ULK1 and LC3. The yellow sphere represents flavokawains, red coloured inhibitory sign indicates suppression/downregulation and green coloured arrow indicates induction/upregulation. Abbreviations: Akt (Protein kinase B), β-catenin (Beta-catenin), CDK (Cyclin-dependent kinase), ERK1/2 (Extracellular signal-regulated kinase 1/2), GSK3β (Glycogen synthase kinase 3 beta), KLF4 (Krüppel-like factor 4), MMP (Matrix metalloproteinase), mTORC2 (Mammalian target of rapamycin complex 2), NF-κB (Nuclear factor kappa-light-chain-enhancer of activated B cells), OCT4 (Octamer-binding transcription factor 4), PI3K (Phosphoinositide 3-kinase), SOX2 (SRY-box transcription factor 2), XIAP (X-linked inhibitor of apoptosis protein).

The major molecular signaling pathways modulated by flavokawains across various cancer types are illustrated in Figure 5.

## 7. Flavokawains and the Tumor Microenvironment (TME)

Flavokawains exert multifaceted regulatory effects within the TME, targeting key processes including angiogenesis and EMT. Therapeutic strategies aimed at reprogramming or disrupting the TME have garnered significant attention in oncological research because the TME plays a pivotal role in tumor initiation, progression, metastasis, and therapeutic resistance. Current therapeutic approaches targeting the TME include anti-angiogenic therapies, like bevacizumab (VEGF inhibitor), which aim to disrupt pathological neovascularization that sustains tumor growth [122]. Immune checkpoint inhibitors, including PD-1/PD-L1 and CTLA-4 antibodies, release inhibitory brakes on cytotoxic T cells, restoring anti-tumor immunity [123]. Evidence from the aforementioned studies indicates that FKA suppresses vasculogenic mimicry and metastatic behavior in HCC by downregulating VE-cadherin, vimentin, Snail1, CXCR4, HIF-1α, NF-κB, and phosphorylated PI3K/Akt, while restoring E-cadherin expression [29]. Similarly, FKC inhibits migration in liver cancer cells by attenuating p-FAK, p-PI3K, and p-Akt signaling and enhances γ-H2AX expression in vivo, indicating impaired survival signaling and increased DNA damage within the tumor niche [48].

Angiogenesis, a key hallmark of TME, is markedly suppressed by flavokawains. For example, synthetic FKA and FKB inhibited angiogenesis in breast cancer models [21,65]. Moreover, FKC treatment significantly downregulated the expression of key pro-angiogenic mediators, including VEGF and Ang-1, in nasopharyngeal carcinoma [79]. Further, FKA modulates the neuroblastoma TME by inactivating ERK signaling, leading to suppression of VEGF-driven angiogenesis, inhibition of MMP2, MMP9, MMP14, and inhibition of EMT-associated migration and invasion, collectively impairing pro-metastatic niche formation [86].

FKB modulates the TME in squamous carcinoma by targeting metastasis- and invasion-associated proteolytic systems. Specifically, treatment with FKB suppressed MMP-9 and u-PA expression and activity, while upregulating their endogenous inhibitors TIMP-1 and PAI-1, thereby limiting extracellular matrix degradation and suppressing invasive niche formation. In vivo xenograft analyses further demonstrated reduced angiogenesis in FKB-treated tumors [94]. FKA significantly downregulated Nanog expression in prostate CSC-derived prostaspheres and xenograft tumors, thereby disrupting the CSC-supportive TME [89]. FKA-A NPs in ovarian cancer downregulated SKP2 expression, thereby impairing SKP2-mediated YAP activation. This led to decreased migratory capacity and suppression of EMT, characterized by upregulation of E-cadherin and downregulation of vimentin, collectively restricting invasion and metastatic progression [87]. In OSCC, FKA and FKB significantly reduced tumor cell migration and invasion without affecting adhesion to extracellular matrix components [76]. 

Beyond their direct effects on tumor cells, flavokawains exhibit notable immunomodulatory properties. In a murine in vivo study, oral administration of FKA and FKB (50 mg/kg/day for 28 days) increased the populations of CD4+/CD3+ T helper cells and CD8+/CD3+ cytotoxic T lymphocytes in splenocytes, with FKB producing a more pronounced effect. This was accompanied by a dose-dependent upregulation of IL-2, a cytokine essential for T-cell proliferation and immune homeostasis [27]. Both compounds suppressed TNF-α levels, reduced NO production, and significantly decreased M2-polarized macrophage populations, with FKB reducing M2 phenotype from 11.6% to 2.2% [27].

Given that M2 tumor-associated macrophages are well-established drivers of tumor progression and immune evasion, the ability of flavokawains to suppress M2 polarization and restore T cell effector populations suggests a potential capacity to modulate immune-related components of the TME. Although direct investigations of flavokawain-mediated immune regulation within the TME remain limited, evidence from a murine model suggests that these compounds possess immunomodulatory properties that may have implications for TME regulation.

Taken together, the available evidence indicates that flavokawains influence multiple interconnected components of the TME rather than acting solely through direct cytotoxic effects on tumor cells. By suppressing angiogenesis, limiting extracellular matrix remodeling, reversing EMT-associated changes, targeting cancer stemness-related pathways, and exhibiting preliminary immunomodulatory activity, flavokawains may alter the biological conditions that support tumor growth, invasion, and metastasis. Although current evidence is derived predominantly from preclinical studies, these collective findings provide a stronger rationale for considering flavokawains as potential modulators of TME reprogramming.

## 8. Toxicity Concerns and Safety Issues

In comparative cytotoxicity analyses conducted in HepG2 cells, the three chalcones were evaluated; among them, FKB exhibited the greatest cytotoxic activity, with an LD_50_ of approximately 15.3 µM, whereas FKA and FKC showed comparatively lower potency, with LD_50_ values of 75 µM and 70 µM, respectively [124]. FKB also induced significant cell death in non-cancerous L-02 hepatocytes. Morphological assessment of L-02 cells following FKB treatment revealed features characteristic of apoptosis, including cell rounding, loss of microvilli, and membrane blebbing. Treatment with FKB at 30 µM in HepG2 cells resulted in caspase-3 activation, further confirming the induction of apoptosis. Additionally, FKB markedly inhibited NF-κB transcriptional activity in HepG2 cells in a dose-dependent manner, as demonstrated by suppression of TNF-α-induced IκB transcription and reporter gene assays. Pre-treatment of HepG2 cells with FKB (30 µM), followed by TNF-α stimulation, led to a significant increase in the phosphorylation of stress-related MAPKs, including JNK, p38, and ERK, suggesting activation of stress response pathways. In L-02 cells, FKB treatment also reduced intracellular GSH levels, indicating oxidative stress. Under in vivo conditions, oral administration of FKB at 25 mg/kg in mice resulted in hepatic injury, evidenced by elevated serum aspartate aminotransferase (AST) and alkaline phosphatase (AKP) levels. Moreover, FKB significantly suppressed LPS-induced NF-κB activation in mouse liver tissue, supporting its immunomodulatory effect [124]. In contrast, a study using TRAMP mice with an FVB/N genetic background found that dietary administration of FKA at 0.6% (*w*/*w*), equivalent to 6 g/kg in the AIN-76A diet, was well tolerated and produced no observable adverse effects on food intake or body weight. Histopathological evaluation demonstrated that FKA exposure did not influence the relative organ weights or morphology of key organs, including the heart, liver, lungs, spleen, kidneys, thymus, colon, and testes. Tissue analysis showed no evidence of pathological changes, including inflammation, fibrosis, atrophy, parenchymal disruption, or epithelial abnormalities. Furthermore, the serum biochemical markers ALT, AST, alkaline phosphatase, creatinine, glucose, and cholesterol remained within physiological limits, indicating preserved hepatic, renal, and systemic function. Additionally, assessment of bone marrow cell colony formation revealed no significant alterations in clonogenic potential following FKA treatment, further supporting its favorable safety profile [125]. The hepatotoxic potential of FKA and FKB was investigated in C57BL/6 mice to assess their individual and combined effects on liver function. When administered separately, FKB (11.5 mg/kg) does not produce any signs of liver toxicity, as evidenced by serum ALT and AST levels remaining within physiological ranges. Moreover, FKA exhibited no toxicity toward normal human hepatic epithelial THLE-3 cells, highlighting its selective anticancer activity and favorable safety profile. However, FKB, when combined with acetaminophen (APAP), resulted in a marked potentiation of APAP-induced liver injury, demonstrated by a significant increase in ALT and AST levels, suggesting a synergistic interaction that enhances hepatocellular damage. Combinatorial treatment of FKA and FKB with APAP resulted in higher ALT and AST levels, resulting in hepatotoxicity [126]. The toxicity profile of flavokawains in different experimental models is summarized in Table 3.

Collectively, the available evidence indicates that the safety profile of flavokawains is both compound- and context-dependent. While FKB has demonstrated hepatotoxic potential under specific experimental conditions and acetaminophen-induced liver injury models, flavokawains administered alone have generally exhibited acceptable tolerability in several in vivo models. Thus, the favorable safety outcomes reported for certain flavokawains should be interpreted within the context of the specific compounds, doses, and experimental models evaluated. Therefore, future pharmacokinetic, dose-escalation, and long-term safety studies are required to more clearly define their therapeutic index and overall safety profile.

## 9. Limitations and Future Perspectives

While this review provides a comprehensive overview of the anticancer potential of flavokawains, certain limitations should be acknowledged. The literature search was conducted primarily using the PubMed database, which, despite its extensive coverage of biomedical research, may not encompass all relevant studies indexed in other databases. Future reviews incorporating multiple databases and broader search strategies would provide a more exhaustive assessment of the available evidence.

Flavokawains have demonstrated significant anticancer potential across various in vitro and in vivo cancer models, primarily through modulation of key molecular pathways involved in proliferation, apoptosis, invasion, migration, angiogenesis, and EMT. However, several limitations hinder their translational application. First, although flavokawains show promising cytotoxic effects, comprehensive toxicity profiling in normal, non-cancerous cells and animal models remains limited. Dose-dependent toxicological studies are essential to evaluate their safety margins and potential off-target effects. Given the growing clinical success of immune checkpoint blockades, future studies should investigate whether flavokawains modulate immune checkpoint pathways, including PD-1/PD-L1, CTLA-4, TIGIT, and LAG-3, and evaluate their potential as adjuvants to immune checkpoint inhibitors.

The clinical utility of flavokawains also requires deeper investigation into their potential roles as chemosensitizing and radiosensitizing agents. Such studies could reveal synergistic effects with conventional therapies and help overcome resistance mechanisms. Furthermore, although a limited number of studies have used flavokawain-based nanoparticles, liposomes, or other delivery systems to improve pharmacokinetics, more extensive, standardized nanocarrier-based studies are needed to optimize targeted delivery and minimize systemic toxicity. Furthermore, the potential for herb–drug interactions involving flavokawains requires comprehensive evaluation in future studies.

Beyond oncology, the therapeutic potential of flavokawain in chronic diseases such as neurological, cardiovascular, and gastrointestinal disorders, like gastritis, which may predispose individuals to cancer, remains underexplored. Investigating their multi-targeted effects in these contexts could broaden their clinical relevance. Future research should include well-designed in vivo studies with dose–response evaluation and long-term safety assessment. Finally, robust clinical trials involving larger and more diverse populations are imperative to establish their pharmacological efficacy, safety, and potential integration into standard treatment regimens.

## 10. Conclusions

Flavokawains have emerged as bioactive chalcone derivatives with significant antineoplastic activity across a range of cancer models in preclinical investigations. Accumulating evidence indicates that these compounds can potentiate the efficacy of established chemotherapeutic agents through synergistic interactions, thereby improving overall therapeutic outcomes. Importantly, flavokawains demonstrate a degree of tumor selectivity, exerting cytotoxic effects on malignant cells while sparing normal tissues to a considerable extent. This differential activity positions them as favorable candidates for drug development. Collectively, these findings support continued exploration of flavokawains as novel phytochemical-based therapeutics, either as standalone agents or in combination regimens, with the potential to achieve enhanced efficacy and reduced systemic toxicity compared to conventional treatments.

## Figures and Tables

**Table 1:** The chemical structures, physicochemical properties, and bioactivities of flavokawains.

Features	Flavokawain A	Flavokawain B	Flavokawain C
Natural source	*Piper methysticum*, *Chloranthus henryi*	*Piper methysticum*, *Alpinia pricei* Hayata, *Polygonum ferrugineum Wedd.*	*Piper methysticum*
Molecular formula	C_18_H_18_O_5_	C_17_H_16_O_4_	C_17_H_16_O_5_
Molecular weight	314.3 g/mol	284.31 g/mol	300.30 g/mol
Melting point	113 °C	91 °C	194–195 °C
Appearance	Yellow crystalline	Yellow crystalline	Yellow crystalline
A-Ring substituents	2′-Hydroxy, 4′6′-Methoxy	2′-Hydroxy, 4′6′-Methoxy	2′-Hydroxy, 4′6′-Methoxy
B-Ring substituents	4-Methoxy	-	4-Hydroxy
Core	α, β-unsaturated ketone	α, β-unsaturated ketone	α, β-unsaturated ketone
Hydroxyl content	1 OH group	1 OH group	2 OH group
Binding	Weak H-bond	No extra H-bond	Strong H-bond
Electrophilicity	Moderate	High	Low
Antioxidant activity	Low	Low	Low
Apoptosis induction	High	High	Moderate
G2/M arrest	Yes	Yes	Yes
Cytotoxicity	Moderate	High	Moderate
Selectivity	High	Moderate	High

**Table 2:** Therapeutic effects of flavokawains on different cancer types.

Cancer Types	Flavokawains	In Vitro/In Vivo/Ex Vivo	Conc./Dosage	Model	Mechanism of Action or Outcome	References
**B-cell lymphoma**	FKB	In vitro	1.25, 2.5, 5, 10, 20 µg/mL	SUDHL-4, Raji, Jeko	↓ Cell viability, p-Akt, p-mTOR, BcL-xL, p-GSK3β; ↑ cleaved PARP, caspase-3	[46]
		In vivo	0.75 mg/kg.b.wt (every 4 days)	SUDHL-4-derived xenograft in nude mice	↓ Tumor weight, Ki-67	
**Bladder Cancer**	FKA	In vitro	10, 20, 40, 60, 80 μM	UMUC3, T24 cells	↓ PRMT5, cell viability, H2AR3, H4R3 methylation; ↑ apoptosis	[55]
		In vivo	30 mg/kg.b.wt (every 3 days)	UMUC3-derived xenograft in nude mice	↓ Tumor size, H2A, H4 R3 methylation	
	FKA + GC	In vitro	10–80 μM	UMUC3, T24 cells	↓ Cell viability	
	FKA	In vivo	6 g/kg in AIN-93M diet	UPII-SV40T transgenic mice	↓ Tumor weight, Ki-67, Bcl-2, XIAP, survivin; ↑ survival of mice, TUNEL-positive cells, DR-5, p27	[56]
	FKA	In vitro	4–40 µM	RT4 cells	↓ Cell growth, CDK2, SKP2; ↑ G1, G2/M phase arrest, p21, p27	[57]
		In vivo	50 mg/kg.b.wt/day	RT4-derived xenograft in nude mice	↓ Tumor growth	
	FKA	In vitro	0.05, 0.5, 5, 12.5, 25 µg/mL	T24 cells	↓ Cell proliferation, survivin, XIAP, Bcl-xL; ↑ caspase-3, -9, Bax	[45]
		In vivo	50 mg/kg.b.wt/day	EJ-derived xenograft in nude mice	↓ Tumor growth	
	FKA + yangonin	In vitro	FKA: 50 µg/mL; Yangonin: 5, 12.5 µg/mL	UMUC3 cells	↓ Cell viability	[58]
**Bone Cancer** Osteosarcoma	FKA	In vitro	2.5, 5, 10, 25, 50 μg/mL	143B, SaOS-2 cells	↓ Cell viability, invasion, SKP2, RhoA, MMP-9; ↑ G2/M arrest, caspase-3, cleaved PARP, p21	[59]
		In vivo	200, 600 mg/kg.b.wt/day	143B-derived xenograft in SCID mice	↓ Lung metastasis, p27, SKP2; ↑ apoptosis	
Synovial sarcoma	FKA	In vitro	2.5, 5, 10, 25 μg/mL	HSSY-II, SYO-I, Yamato, Aska cells	↓ Cell viability, invasion, SKP2, ETV4, ECT2, vimentin, Twist, ZO-1, sarcosphere; ↑ apoptosis, G2/M arrest, E-cadherin, cleaved PARP, p27	[60]
		In vivo	600 mg/kg.b.wt/day	HSSY-II-derived xenograft in SCID mice	↓ Tumor growth	
	FKB	In vitro	2.5, 5, 7.5 μg/mL	SYO-I, HSSY-II cells	↓ Cell growth, colony formation, IAP, survivin, Bcl-2; ↑ caspase-3, -7, -8, -9, DR5, Bim, Puma, Bax	[61]
**Breast Cancer**	13, 15, 16 (FKB derivatives)	In vitro	0.47–30 µg/mL	MCF-7, MDA-MB-231 cells	↑ Cytotoxicity	[62]
	FKA	In vitro	2, 4, 8, 16 μM	SKBR3, MCF-7, MDA-MB-468 cells	↓ Cell growth, colony formation, HER2, p-Akt, Bcl-2, survivin, XIAP; ↑ G2/M arrest, Cdc2, apoptosis, Bim, Bax, cleaved PARP	[63]
	FLS (FK derivative)	In vitro	3–180 μM	MCF-7, MDA-MB-231 cells	↓ Cell viability, Bcl-2, Cdc2; ↑ G2/M arrest, apoptosis, caspase-9, cytochrome c, Bax, p53, c-Jun, WEE-1	[64]
	FKB (synthetic)	In vitro	6–40.5 μM	MDA-MB-231 cells	↓ Cell proliferation, migration, invasion, angiogenesis; NF-κB, COX-2 ↑ G2/M arrest, apoptosis	[65]
		Ex vivo	6, 12.3, 24.6 μM	Male Sprague Dawley rats	↓ Angiogenesis	
	FKA (synthetic)	In vitro	6.5–70 μM	MCF-7, MDA-MB-231 cells	↓ Cell proliferation, PLK1, FOXM1; ↑ apoptosis, G2/M arrest, caspase-8, -9, Bax, cytochrome c	[21]
		Ex vivo	6.5, 17.5, 65 μM	Male Sprague Dawley rats	↓ Angiogenesis	
**Cervical Cancer**	FKB	In vitro	1.56–100 µM	HeLa cells	↑ Cytotoxicity, G2/M arrest, apoptosis, SOD2, GSH, cytochrome C, p-p38α, p-HSP27, HSP70	[66]
**Cholangiocarcinoma**	FKB; cisplatin; FKB + cisplatin	In vitro	0–100 μM	SNU-478	↓ Cell viability, p-Akt; ↑ apoptosis, cleaved PARP	[22]
	FKB; FKB + cisplatin/gemcitabine	In vivo	Cisplatin: 5 mg/kg, gemcitabine: 100 mg/kg and FKB: 25 mg/kg (twice a week)	SNU-478-derived xenograft in nude mice	↓ Tumor growth	
**Colorectal Cancer**	FKA	In vitro	25,100, 200, 500 μg/mL	SW620, DLD-1, HT-29, HCT-8, HCT-116 cells	↓ Cell viability	[67]
	FKC	In vivo	1, 3 mg/kg.b.wt (thrice weekly)	HCT-116-derived xenograft in nude mice	↓ Tumor growth, Ki-67; ↑ apoptosis, cleaved caspase-3	[68]
	FKB	In vitro	10, 20 μM	LoVo, LoVo/Dx cells	↓ Cell growth, PCNA; ↑ apoptosis, G2/M arrest	[69]
	FKC	In vitro	60 μM	HCT-116 cells	↓ MLC-3, GAMT, DCK, PGAM1, GSTO1, COMT, BPNT1, CNPY2, TCEB1, CRABP2, eIF-5A, EEF-2, eIF-3I, CBX3, SKP1, ATP5H, RanBP1, hTom22; ↑ Hspa8, Hsp70-Hom, Hsp27, Hsp70-1/Hsp70-2, Hsp86, TCP-1-eta, CK-18, Tubulin beta-2 chain, Gamma actin, P4HB, HMOX1, FKBP4, HR23B, SFPQ, PARK7, GLOD4, GLRX3	[70]
	FKC	In vitro	40, 60, 80 µM	HCT-116 cells, HT-29	↓ Cell viability, SOD; ↑ apoptosis, caspase-3-, -8-, -9-positive cells, cleaved PARP-1, ROS, p21, p27, G2/M arrest	[71]
	FKB	In vitro	0.1–50 μM	HCT-116 cells	↓ Cell viability, proliferation, colony formation, Bcl-2; ↑ apoptosis, cleaved PARP, GADD153, Bim L, Bim S, p-38 MAPK, ROS, G2/M arrest, autophagy	[72]
	FKA	In vivo	20, 80 mg/kg/2 days	AOM/DSS-induced CRC in mice	↓ Tumor count, polyp size, LPS biosynthesis pathway	[73]
**Gastric Cancer**	FKB + doxorubicin	In vitro	FKB: 1.25, 2.5, 5 µg/mL; Dox: 0.5 µg/mL	AGS, SCM-1, MKN-45	↓ ATG4B; ↑ autophagy, apoptosis, caspase-3, -8, -9, Fas-FasL, LC3-II, Beclin-1, ROS	[74]
		In vivo	FKB: 0.75 mg/kg.b.wt; Dox: 1.5 mg/kg.b.wt (every 2 days)	AGS-derived xenograft in nude mice	↓ Tumor growth	
	FKB	In vitro	2.5–20 μg/mL	AGS, NCI-N87, Hs738, Kato-III, TSGH-9201 cells	↓ Cell survival, colony formation, ATG4B, HER2, PI3K, Akt, mTOR; ↑ LC3-II, autophagy, ROS, apoptosis, JNK, ERK, G2/M arrest, cyclin A, cyclin B1, CDK1, Cdc25C	[49]
		In vivo	1.5, 7.5 mg/kg.b.wt (every 2 days)	AGS-derived xenograft in nude mice	↓ Tumor growth	
	FKB	In vitro	10 μg/mL	SGC-7901	↓ Cell growth, proliferation, Cdc2, Cdc25C, cyclin A, cyclin B1, CCR2, macrophage migration; ↑ G2/M arrest, apoptosis, caspase-3, -7, -8, -9, TSPAN12, TGF-β1, SMAD4	[75]
		In vivo	1.5 mg/kg.b.wt (every 2 days)	SGC-7901-derived xenograft in nude mice	↓ Tumor weight; ↑ SMAD4, TGF-β1, TSPAN12, survival time	
**Head and Neck Cancers** Oral squamous cell carcinoma	FKA, FKB	In vitro	10 μg/mL, 2.5 μg/mL	H400, BICR56, OKF6 cells	↓ Cell growth, migration, invasion	[76]
Oral carcinoma	FKB	In vitro	1.25–10 μg/mL; 4.4–35.2 μM	HSC-3, Cal-27 cells	↓ Cell viability, cyclin A, cyclin B, Cdc2, Cdc25C, Bcl-2, PI3K/Akt, p38 MAPK; ↑ G2/M arrest, apoptosis, cytochrome c, Bax	[77]
Oral adenoid cystic carcinoma	FKB (synthetic)	In vitro	1.1, 2.2, 4.4, 8.8, 17.6, 44, 87.9 µmol/L	ACC-2 cells	↓ Cell growth, Bcl-2; ↑ apoptosis, G2/M arrest, Bim, Bak, Bax	[78]
Nasopharyngeal carcinoma	FKC	In vitro	0.5, 1, 2, 4 μM	HNE1, HNE2, CNE1, CNE2, HONE1 cells	↓ HSP90B1, Ang-1, VEGF, EGFR/PI3K/Akt/mTOR	[79]
		In vivo	3 mg/kg.b.wt	HNE1-derived xenograft in nude mice	↓ Tumor volume, tumor weight, HSP90B1, GLUT1, HK2, Ang-1, VEGF, EGFR/PI3K/Akt/mTOR	
**Liver Cancer**	FKA	In vitro	10, 20, 40 μM	SMMC-7721, huh7, PANC-1, HepG2, HeLa, Hep3B, A549 cells	↓ Cell viability, migration, invasion, CXCR4, proliferation, VM formation, VE-cadherin, vimentin, Snail1, EMT, p-PI3K, p-Akt, HIF-1α, NF-κB, Twist1; ↑ apoptosis, E-cadherin	[29]
		In vivo	30, 60, 120 mg/kg.b.wt/day	HepG2-derived xenograft in nude mice	↓ Tumor growth, EMT, metastasis, VE-cadherin, vimentin, Twist1, p-Akt; ↑ E-cadherin	
	FKA	In vitro	2–100 μM	HepG2 cells	↓ Cell viability; ↑ toxicity, Nrf2, HSF1, HMOX1, GCLC, HSPA1A, DNAJA4, GSH	[50]
	FKB	In vitro	2–100 μM	HepG2 cells	↓ Cell viability; ↑ toxicity, Nrf2, HSF1, HMOX1, GCLC, HSPA1A, DNAJA4, GSH	
	FKC	In vitro	4, 8, 16 μM	Huh-7, Hep3B, HepG2 cells	↓ Cell proliferation, migration, Bax, Bcl-2, p-FAK, p-PI3K, p-Akt; ↑ apoptosis	[48]
		In vivo	16 mg/kg.b.wt/day	Huh-7-derived xenograft in nude mice	↓ Tumor growth, Ki-67; ↑ γ-H2AX	
**Lung Cancer**	FKA	In vitro	5–30 µM	A549, PTX-resistant A549 (A549/T), THLE-3 cells	↓ Cell viability, cell growth, P-gp, Akt, p-Akt; ↑ apoptosis, PARP	[80]
	FKA, FKB, FKC	In vivo	FKA, FKB: 5 mg/g, FKC: 2.5 mg/g.b.wt	NNK, BaP-induced tumor in mice	↓ Tumor multiplicity	[51]
	Chalcone-24 (FK synthesized)	In vitro	0.3, 1, 3 μM	A549 cells	↓ Cell viability, NF-κB; ↑ ERK1/2, JNK, caspase activity	[81]
	FKB	In vitro	5–15 μg/mL	A549, H1299 cells	↓ Cell viability, colony formation, Bcl-2; ↑ caspase-3, -9, Bax, LC3	[82]
**Leukemia** Acute Myeloid Leukemia (AML)	FKA	In vitro	2.5, 5, 10, 20 μg/mL	MV4-11, THP-1, MOLM-13, U937 cells	↓ Cell viability, CDT1, CCND1, CCNE1, CCNE2, CDK2, CDK4, CDK6; ↑ p27, G1 arrest, CDKN1B	[83]
		Ex vivo	2.5, 5, 10, 20 μg/mL	Primary AML blasts from patients	↓ Cell viability	
Acute Lymphoblastic Leukemia (ALL)	FKB	In vitro	0.1–120 μM	CCRF-CEM, CEM-C1, Jurkat, RS4-11	↓ Cell viability; ↑ apoptosis, caspase-3, cleaved PARP, p53, Bax, Puma	[52]
		In vivo	0.75 mg/kg.b.wt/day	CCRF-CEM-inoculated Balb/c mice	↓ Leukocytes, WBC, splenomegaly	
		Ex vivo	25–100 μM	Primary B-ALL blasts, T-ALL blasts	↓ Cell proliferation; ↑ p53, Bax, Puma	
Myeloid Leukemia	FKB	In vitro	0.5–24 µM	HL-60, K562, MOLT4, CEM1 cells	↓ Cell viability; ↑ apoptosis	[84]
	FKB + Daunorubicin (DNR)	In vitro	FKB: 10, 12, 2, 3 µM; DNR: 0.15, 0.05, 0.005, 0.01 µM	HL-60, K562, MOLT4, CEM1 cells	↓ Cell viability; ↑ NF-ĸB	
**Melanoma**	FKA, FKB (synthetic)	In vitro	1.56, 6.25, 25 µM	B16/F10 cells	↓ Cell viability, cellular melanin, Tyr, Trp-1, Trp-2, Mitf	[85]
		In vivo	FKA: 0.78–25 µM, 25 µM; FKB: 0.78–12.5 µM, 6.25 µM	Zebrafish	↓ Melanin production; ↑ survival rate	
**Neuro-oncological malignancies** Glioblastoma	FKB	In vitro	1–5 μg/mL	U251, U87, T98 cells	↓ Cell viability, proliferation, SQSTM1, p-mTOR, p-Akt, p-RPS6KB1; ↑ G2/M arrest, senescence, autophagy, MAP1LC3B-II, HSPA5, p-EIF2AK3, p-EIF2A, ATF4, DDIT3, p-γH2AX	[53]
		In vivo	50 mg/kg.b.wt/day	U251-derived xenograft in nude mice	↓ Tumor growth	
Neuroblastoma	FKA	In vitro	12.5, 25, 50 μM	SK-N-SH, HUVEC cells	↓ Cell viability, colony formation, migration, invasion, angiogenesis, N-cadherin, Snail, VE-cadherin; ↑ apoptosis, E-cadherin, G1 arrest	[86]
**Ovarian Cancer**	FKA-A NPs+ PTX-A NPs	In vitro	FKA-A NPs: 2.5–60 μM); PTX-A NPs: 2.5–60 μM	A2780, SKOV-3 cells	↓ Cell proliferation, colony formation, migration, vimentin, SKP2, nuclear translocation of YAP; ↑ E-cadherin	[87]
		In vivo	PTX-A NPs + FKA-A NPs: 2.5 + 2.5 mg/kg.b.wt (every 3 days)	A2780-derived xenograft in nude mice	↓ Tumor growth	
	FKC	In vitro	1–100 µM	SKOV-3, SW-626, OVCAR3, CaOV-3, IOSE80	↓ Cell viability	[88]
**Prostate Cancer**	FKA	In vitro	5, 12.5, 25 µM	22Rv1, DU145, CD44^+^/CD133	↓ Tumor spheroid size, number, Oct4, Sox2, Nanog, c-Myc, CK8, Ubc12 neddylation	[89]
		In vivo	6 g/kg FKA in diet	CD44^+^/CD133^+^ 22Rv1-derived xenograft in NOD/SCID mice	↓ Tumor growth, Ki-67, c-Myc, Ubc12 neddylation, Nanog	
	FKA	In vitro	1.56, 3.12, 6.25, 12.5, 25, 50, 100 µM	PC3 cell line	↓ Cell proliferation, survivin, GSH, GSS; ↑ apoptosis, G2/M arrest, GSTP1, ROS	[90]
	FKB	In vitro	8.8 μM	C4-2B, PC3	↓ SKP2; ↑ p27/Kip1	[91]
	FKB	In vitro	1.1, 2.2, 4.4, 8.8, 17.6 µM	LNCaP, LAPC4, DU145, PC3	↓ Cell viability, XIAP, survivin; ↑ apoptosis, caspase-3, -8, -9, DR5, Bim, Puma	[54]
		In vivo	50 mg/kg.b.wt/day	DU145-derived xenograft in nude mice	↓ Tumor growth; ↑ Bim	
	FKA	In vitro	4–80 µM	DU145 (*Rb^−/−^*), PC3 (*Rb^+/+^*), 22Rv1 (*Rb^+/+^*), MEF (*Rb^+/+^*, *Rb^−/−^*), MPEC (*Rb^+/+^*, *Rb^−/−^*) cells	↓ Cell growth, SKP2, Cullin-1, Ubc12 neddylation	[92]
		In vivo	3, 6 g/kg FKA in diet	TRAMP mice	↓ Cell proliferation, HG-PIN, prostate adenocarcinoma, tumor burden, metastasis, Ki-67, SKP2, NEDD8; ↑ apoptosis, p27	
	FKB	In vitro	0.63–25 μg/mL	LNCaP, LAPC4, 22Rv1, PC3, DU145, WPMY-1 cells	↓ Cell growth, AR, PSA, TMPRSS2	[93]
		In vivo	200 mg/kg.b.wt/day	Patient-derived xenograft in SCID mice	↓ Tumor growth, PSA, AR	
**Squamous carcinoma**	FKB	In vitro	5, 10, 20 μg/mL	KB cells	↓ Cell viability, Bcl-2, cyclin A, cyclin B1, Cdc2, Cdc25C, MMP-9, uPA; ↑ apoptosis, G2/M arrest, caspase-3, -9, Bax, p21/WAF1, Wee1, p53, TIMP-1, PAI-1	[94]
		In vivo	0.75 mg/kg.b.wt (every 2 days)	KB-induced xenograft in nude mice	↓ Tumor volume, angiogenesis; ↑ TUNEL-positive cells	
**Uterine leiomyosarcoma**	FKB	In vitro	1.1, 2.2, 4.4, 8.8 μM	SK-LMS-1, ECC-1, T-HESC cells	↓ Cell growth, IAP, survivin; ↑ G2/M arrest, DR5, Bim, Puma	[95]
	FKB + Docetaxel + Gemcitabine	In vitro	FKB: 2.2, 4.4 μM; Docetaxel + Gemcitabine: IC_50_	SK-LMS-1 cells	↓ Cell proliferation	

Symbols and Abbreviations: ↓ (Downregulated/Decreased), ↑ (Upregulated/Increased); Akt (Protein kinase B), AML (acute myeloid leukemia), Ang-1 (Angiopoietin-1), AR (androgen receptor), ATF4 (Activating Transcription Factor 4), ATG4B (Autophagy-Related 4B Cysteine Peptidase), AOM (Azoxymethane), BaP (Benzo[a]pyrene), Bax (Bcl-2-associated X protein), Bcl-2 (B-cell lymphoma 2), Bcl-xL (B-cell lymphoma-extra large), Bim (Bcl-2-interacting mediator of cell death), BPNT1 (3′(2′),5′-Bisphosphate Nucleotidase 1), CBX3 (Chromobox Protein Homolog 3), CCR2 (C-C Motif Chemokine Receptor 2), CDK (Cyclin-Dependent Kinase), CDT1 (Chromatin Licensing and DNA Replication Factor 1), CK8 (Cytokeratin 8), COMT (Catechol-O-Methyltransferase), CNPY2 (Canopy FGF Signaling Regulator 2), COX-2 (Cyclooxygenase-2), CRABP2 (Cellular Retinoic Acid-Binding Protein 2), CXCR4 (C-X-C Motif Chemokine Receptor 4), DCK (Deoxycytidine Kinase), DDIT3 (DNA Damage-Inducible Transcript 3), DNR (Daunorubicin), DR5 (Death Receptor 5), DSS (Dextran Sulfate Sodium), EF-2 (Elongation Factor 2), EGFR (Epidermal Growth Factor Receptor), EIF2AK3 (Eukaryotic Translation Initiation Factor 2 Alpha Kinase 3/PERK), EIF-3I (Eukaryotic Translation Initiation Factor 3 Subunit I), EIF-5A1 (Eukaryotic Translation Initiation Factor 5A1), EMT (epithelial-mesenchymal transition), ERK1/2 (Extracellular Signal-Regulated Kinase 1/2), ETV4 (ETS Variant Transcription Factor 4), ECT2 (Epithelial Cell Transforming Sequence 2), FAK (Focal Adhesion Kinase), FasL (Fas Ligand), FKA (Flavokawain A), FKB (Flavokawain B), FKC (Flavokawain C), FKA-A NPs (Flavokawain A-loaded nanoparticles), FLS (Flavokawain-like Synthetic Derivative), FOXM1 (Forkhead Box M1), GADD153 (Growth Arrest and DNA Damage-Inducible Protein 153/CHOP), GAMT (Guanidinoacetate N-Methyltransferase), GCLC (Glutamate-Cysteine Ligase Catalytic Subunit), GLUT1 (Glucose Transporter 1), GLOD4 (Glyoxalase Domain Containing 4), GLRX3 (Glutaredoxin 3), GSH (Glutathione), GSSG (Oxidized Glutathione), GSTO1 (Glutathione S-Transferase Omega 1), GSTP1 (Glutathione S-Transferase Pi 1), HER2 (Human Epidermal Growth Factor Receptor 2), HIF-1α (Hypoxia-Inducible Factor 1 Alpha), HK2 (Hexokinase 2), HMOX1 (Heme Oxygenase 1), HSP27 (Heat Shock Protein 27), HSP70 (Heat Shock Protein 70), HSP86 (Heat Shock Protein 86), HSP90B1 (Heat Shock Protein 90 Beta Family Member 1), HSF1 (Heat Shock Factor 1), HR23B (UV Excision Repair Protein RAD23 Homolog B), IAP (Inhibitor of Apoptosis Protein), JNK (c-Jun N-terminal Kinase), LC3-II (Microtubule-associated Protein 1 Light Chain 3-II), LPS (lipopolysaccharide), MAPK (Mitogen-Activated Protein Kinase), MAP1LC3B-II (Microtubule-Associated Protein 1 Light Chain 3 Beta-II), MLC-3 (Myosin Light Chain 3), MMP-9 (Matrix Metalloproteinase-9), mTOR (Mammalian Target of Rapamycin), NF-κB (Nuclear Factor Kappa B), NEDD8 (Neural Precursor Cell-Expressed Developmentally Downregulated Protein 8), Nrf2 (Nuclear Factor Erythroid 2-Related Factor 2), Oct4 (Octamer-Binding Transcription Factor 4), PARP (Poly(ADP-ribose) Polymerase), PAI-1 (Plasminogen Activator Inhibitor-1), PARK7 (Parkinsonism-Associated Deglycase), PCNA (Proliferating Cell Nuclear Antigen), P-gp (P-glycoprotein), PGAM1 (Phosphoglycerate Mutase 1), PI3K (Phosphoinositide 3-Kinase), PLK1 (Polo-Like Kinase 1), PRMT5 (Protein Arginine Methyltransferase 5), PSA (Prostate-Specific Antigen), PTX (Paclitaxel), PTX-A NPs (Paclitaxel-loaded Nanoparticles), Puma (p53 Upregulated Modulator of Apoptosis), RanBP1 (Ran Binding Protein 1), RhoA (Ras Homolog Family Member A), ROS (Reactive Oxygen Species), RPS6KB1 (Ribosomal Protein S6 Kinase B1), SKP2 (S-phase Kinase-associated Protein 2), SMAD4 (SMAD Family Member 4), Snail (Snail Family Transcriptional Repressor 1), Sox2 (SRY-Box Transcription Factor 2), SOD (Superoxide Dismutase), TCP-1-eta (T-Complex Protein 1 Eta Subunit), TCEB1 (Transcription Elongation Factor B Subunit 1), TGF-β1 (Transforming Growth Factor Beta 1), TIMP-1 (Tissue Inhibitor of Metalloproteinases-1), TMPRSS2 (Transmembrane Protease Serine 2), Trp-1 (Tyrosinase-Related Protein 1), Trp-2 (Tyrosinase-Related Protein 2), TSPAN12 (Tetraspanin 12), TUNEL (Terminal Deoxynucleotidyl Transferase dUTP Nick End Labeling), Tyr (Tyrosinase), Ubc12 (Ubiquitin-Conjugating Enzyme E2 M), uPA (Urokinase-Type Plasminogen Activator), VEGF (Vascular Endothelial Growth Factor), VM (vasculogenic mimicry), WAF1 (Wild-Type p53-Activated Fragment 1/CDKN1A), WBC (white blood cell), Wee1 (WEE1 G2 Checkpoint Kinase), XIAP (X-linked Inhibitor of Apoptosis Protein), YAP (Yes-Associated Protein), ZO-1 (Zonula Occludens-1), and γ-H2AX (Phosphorylated Histone H2AX), b.wt (body weight).

**Table 3:** Toxicity profile of flavokawains.

Toxicity Type	Flavokawain	In Vitro/In Vivo	Model	Conc./Dosage	Toxicity	References
Hepatotoxicity	FKB	In vitro	HepG2	10–50 μM	↑ Cell death, apoptosis, MAPK, caspase-3; ↓ NF-kB FKB: LD_50_: 15.3 ± 0.2 μM	[124]
	FKB, FKC	In vitro	L-02	0–150 μM	↑ Cell death, apoptosis; ↓ GSH FKB: LD_50_: 32 μM FKC: LD_50_: 70 μM	
	FKA	In vitro	HepG2	0–150 μM	FKA: LD_50_: 75 μM	
	FKB	In vivo	ICR mice	25 mg/kg.b.wt	↑ Liver damage, AST, AKP; ↓ NF-kB	
Hepatotoxicity	FKB	In vivo	C57BL/6J mice (acetaminophen-induced)	FKB: 11.5 mg/kg.b.wt; APAP: 800 mg/kg.b.wt	↑ AST, ALT	[126]
	FKA + FKB	In vivo	C57BL/6J mice	1× dose FKA-8 mg/kg FKB-11.5 mg/kg	No effect on AST and ALT levels	
				2× dose FKA-16 mg/kg FKB-23 mg/kg		
				4× dose FKA-32 mg/kg FKB-46 mg/kg		
	FKA + FKB	In vivo	C57BL/6J mice (acetaminophen-induced)	1× dose FKA-8 mg/kg FKB-11.5 mg/kg	↑ AST, ALT	
				2× dose FKA-16 mg/kg FKB-23 mg/kg		
				4× dose FKA-32 mg/kg FKB-46 mg/kg		
Bone marrow toxicity	FKA	In vitro	Mouse bone marrow cells	0–25 μg/mL	No significant effect on colony formation	[125]
General toxicity	FKA	In vivo	FVB/N mice	6 g/kg in diet	No adverse effects on body weight, food intake, organ histology, or serum biochemistry	
Hepatotoxicity	FKA	In vivo	FVB/N mice	6 g/kg in diet	No increase in liver weight, no histopathological changes, no change in ALT, AST, ALP, albumin	

Symbols and Abbreviations: ↓ (Downregulated/Decreased), ↑ (Upregulated/Increased); FKA, Flavokawain A; FKB, Flavokawain B; FKC, Flavokawain C; LD50, median lethal dose; AST, aspartate aminotransferase; ALT, alanine aminotransferase; ALP/AKP, alkaline phosphatase; LD50, median lethal dose; NF-κB, Nuclear factor kappa B; MAPK, Mitogen-activated protein kinase; GSH, glutathione; ICR mice, Institute of Cancer Research mice.

## Data Availability

No new data were created or analyzed in this study. Data sharing is not applicable to this article.

## Abbreviations

AKP	Alkaline phosphatase
Akt	Protein kinase B
ALT	Alanine aminotransferase
Ang-1	Angiopoietin-1
AP-1	Activator protein 1
APAP	Acetaminophen
AR	Androgen receptor
AST	Aspartate aminotransferase
BaP	Benzo[a]pyrene
Bax	BCL2-associated X protein
BBB	Blood–brain barrier
Bcl-2	B-cell lymphoma 2 protein
Bcl-xL	B-cell lymphoma-extra large
CDK	Cyclin-dependent kinase
COX-2	Cyclooxygenase-2
CSC	Cancer stem cell
DR5	Death receptor 5
EGFR	Epidermal growth factor receptor
EMT	Epithelial–mesenchymal transition
ER	Endoplasmic reticulum
ERK	Extracellular signal-regulated kinase
FAK	Focal adhesion kinase
FKA	Flavokawain A
FKB	Flavokawain B
FKC	Flavokawain C
FOXM1	Forkhead box protein M1
GADD153	Growth arrest and DNA damage-inducible protein 153
GBM	Glioblastoma
GLUT1	Glucose transporter 1
GSH H&E	Glutathione Hematoxylin and Eosin
HCC	Hepatocellular carcinoma
HER2	Human epidermal growth factor receptor 2
HIF-1α	Hypoxia-inducible factor 1-alpha
HK2	Hexokinase 2
HMOX1	Heme oxygenase-1 gene
HSP90B1	Heat shock protein 90 beta family member 1
iNOS	Inducible nitric oxide synthase
JNK	c-Jun N-terminal kinase
LC3-II LD_50_	Microtubule-associated protein 1A/1B-light chain 3-II (autophagy marker) Median lethal dose
LPS	Lipopolysaccharide
MAPK	Mitogen-activated protein kinase
MMP	Matrix metalloproteinase
mTOR	Mammalian target of rapamycin
NF-κB	Nuclear factor kappa-light-chain-enhancer of activated B cells
NNK	4-(Methylnitrosamino)-1-(3-pyridyl)-1-butanone (tobacco carcinogen)
NO	Nitric oxide
Nrf2	Nuclear factor erythroid 2-related factor 2
OSCC	Oral squamous cell carcinoma
PAI-1	Plasminogen activator inhibitor-1
PARP	Poly (ADP-ribose) polymerase
PCNA	Proliferating cell nuclear antigen
PI3K	Phosphoinositide 3-kinase
PLK1	Polo-like kinase 1
PPARγ	Peroxisome proliferator-activated receptor gamma
PRMT5	Protein arginine methyltransferase 5
PSA	Prostate-specific antigen
ROS SA-β-gal	Reactive oxygen species Senescence-associated β-galactosidase
SCID	Severe combined immunodeficiency
Skp2	S-phase kinase-associated protein 2
SOD	Superoxide dismutase
TGF-β1	Transforming growth factor beta 1
TIMP-1	Tissue inhibitor of metalloproteinase-1
TME	Tumor microenvironment
TRAMP	Transgenic adenocarcinoma of mouse prostate
TUNEL	Terminal deoxynucleotidyl transferase dUTP nick end labeling
u-PA	Urokinase-type plasminogen activator
VEGF	Vascular endothelial growth factor
XIAP	X-linked inhibitor of apoptosis protein
